# Efficient disruption of Zebrafish genes using a Gal4-containing gene trap

**DOI:** 10.1186/1471-2164-14-619

**Published:** 2013-09-14

**Authors:** Jorune Balciuniene, Danielle Nagelberg, Kathleen T Walsh, Diana Camerota, Daphné Georlette, Frédéric Biemar, Gianfranco Bellipanni, Darius Balciunas

**Affiliations:** 1 Department of Biology, College of Science and Technology, Temple University, Philadelphia, PA 19122, USA; 2 Sbarro Institute for Cancer Research and Molecular Medicine, Temple University, Philadelphia, PA 19122, USA

**Keywords:** Zebrafish, Insertional mutagenesis, Gene trap, Gal4, Tol2, nsfa, Fleer, atp1a3a, bbs7

## Abstract

**Background:**

External development and optical transparency of embryos make zebrafish exceptionally suitable for *in vivo* insertional mutagenesis using fluorescent proteins to visualize expression patterns of mutated genes. Recently developed Gene Breaking Transposon (GBT) vectors greatly improve the fidelity and mutagenicity of transposon-based gene trap vectors.

**Results:**

We constructed and tested a bipartite GBT vector with Gal4-VP16 as the primary gene trap reporter. Our vector also contains a UAS:eGFP cassette for direct detection of gene trap events by fluorescence. To confirm gene trap events, we generated a UAS:mRFP tester line. We screened 270 potential founders and established 41 gene trap lines. Three of our gene trap alleles display homozygous lethal phenotypes ranging from embryonic to late larval: *nsf* ^*tpl6*^, *atp1a3a*^*tpl10*^ and *flr*^*tpl19*^. Our gene trap cassette is flanked by direct loxP sites, which enabled us to successfully revert *nsf* ^*tpl6*^, *atp1a3a*^*tpl10*^ and *flr*^*tpl19*^ gene trap alleles by injection of Cre mRNA. The UAS:eGFP cassette is flanked by direct FRT sites. It can be readily removed by injection of Flp mRNA for use of our gene trap alleles with other tissue-specific GFP-marked lines. The Gal4-VP16 component of our vector provides two important advantages over other GBT vectors. The first is increased sensitivity, which enabled us to detect previously unnoticed expression of *nsf* in the pancreas. The second advantage is that all our gene trap lines, including integrations into non-essential genes, can be used as highly specific Gal4 drivers for expression of other transgenes under the control of Gal4 UAS.

**Conclusions:**

The Gal4-containing bipartite Gene Breaking Transposon vector presented here retains high specificity for integrations into genes, high mutagenicity and revertibility by Cre. These features, together with utility as highly specific Gal4 drivers, make gene trap mutants presented here especially useful to the research community.

## Background

Amenability to mutagenesis combined with optical transparency of externally developing embryos and large clutch size make zebrafish an excellent model system for developmental genetics [1]. Two large-scale ethylnitrosourea (ENU) mutagenesis screens have clearly established that all aspects of zebrafish development can be analyzed by forward genetics [2-4]. Indeed, these large-scale screens continue to be followed up by more specialized screens for defects in specific biological pathways (reviewed in [5]). High efficiency and random nature of chemical mutagenesis enables generation of multiple alleles of variable strength, as recently reported for *tbx2b*[6,7]. Constant improvements in both mapping resources and fidelity of the assembly of the zebrafish genome have enabled identification of a significant subset of genes affected by chemically-induced mutations. Nonetheless, some chemical mutants of exceptional biological interest remain to be cloned or confirmed. A classic example is the hemangioblast mutant *cloche*, for which only a candidate gene has been reported to date [8,9]. Other examples are still-uncloned cocaine addiction mutants *dumbfish*, *jumpy* and *goody*-*two*-*shoes*[10]. Thus, while chemical mutagenesis can readily generate mutants in biological pathways of interest, it does not always lead to molecular identification of the affected genes.

Insertional mutagenesis is not as random as chemical mutagenesis and does not possess as high efficiency. However, these deficiencies are offset by more straightforward identification of affected genes using the insertional mutagen as a molecular tag. Furthermore, it is possible to use fluorescent reporters to monitor the expression of mutated genes as well as design the insertional mutagen for additional utility. The only insertional mutagen used to date in large scale in zebrafish is the pseudotyped retrovirus. The virus has been used in two complimentary approaches. The first was to mutate genes required for embryonic development, leading to identification of over 330 such genes by a single laboratory [11-13]. The second approach was to analyze the regulatory landscape of the zebrafish genome through enhancer trapping [14]. Albeit successful, retrovirus as an insertional mutagenesis vector has several limitations. First, production and handling of viral particles requires special expertise and facilities. Second, modifications such as addition of gene trap components may result in lower virus titers and require significant optimization of production procedures [15]. Finally, the only retroviral approach that produced fluorescent protein expression-tagged integration events -the enhancer trap- did not produce a significant number of loss-of-function alleles [14].

Success of transposon-based mutagenesis in *Drosophila* (reviewed in [16]) prompted investigation of the activity of different transposons – *Tc1*, *Sleeping Beauty* and *Tol2*- in the zebrafish [17-19]. In contrast to the retrovirus, transposable elements do not possess the machinery to deliver exogenous DNA into the nucleus, which results in somewhat lower rates of integration into the genome and subsequent germline transmission of transposition events. Transposon-based insertional mutagenesis vectors used in zebrafish fall under the general categories of enhancer trap, 5’ gene trap and 3’ gene trap, and include fluorescent reporters to detect “trapping” events. Enhancer and 5’ gene trap vector integrations reveal the expression profile of the tagged locus and are ideal for the optically transparent, externally developing zebrafish embryos. The drawback of enhancer trap vectors is that they usually are not mutagenic and only induce mutations by integrations into exons or other essential sequences of genes (reviewed in [16]). As expected, transposon-based enhancer trap integrations did not result in overt embryonic phenotypes in zebrafish [20,21]. Initial zebrafish gene trap vectors suffered from poor mutagenicity as well [22]. Two reasons may underlie this lack of mutagenicity. First, the vector used in these studies was later found to also function as an enhancer trap [23]. Second, when integration did occur into a gene, the splice acceptor and polyadenylation/transcriptional termination sequences were leaky, allowing for read-through transcription and splicing [22,24]. This leakiness may be partly attributed to use of rabbit β-globin splice acceptor (SA) [22], as there appear to be significant differences between mammalian and fish splice sites [25]. To reduce this read-through transcription and splicing, Sivasubbu and colleagues used fish-derived SA and transcriptional termination/polyadenylation (p(A)) sequences. Integrations into genes were selected using the 3’ gene trap paradigm and enabled identification of the first transposon-induced phenotypic mutation in zebrafish, leading to adaptation of the term “*G*ene *B*reaking *T*ransposon” (GBT) [26]. Notably, GBTs are capable of inducing mutations by integration into introns of genes. This makes GBT-induced mutations reversible by removing the SA/p(A) components.

The next step was to develop a true 5’ gene trap vector by flanking it with a fish-derived and potentially mutagenic SA/p(A) sequences. To make selection for integration into protein-coding genes more stringent, the AUG translation initiation codon of the fluorescent reporter was removed. The 5’ gene trap cassette was flanked by loxP sites, leading to generation of first revertible mutants in zebrafish [27,28]. An alternative mutagenesis strategy relying on integration of trap vectors into exons or other important sequences has also been proposed [29,30]. In parallel, Gal4-based transcriptional activators were being adapted for insertional mutagenesis [23,31,32]. The main advantage of Gal4-based transcriptional activators is that gene- or enhancer-trap lines can be used as drivers for expression of other transgenes including fluorescent reporters, toxin genes, calcium sensors and optically activated channel proteins, in a tissue-specific manner [23,31,33-36].

In this study, we provide proof-of-principle demonstration that a gene breaking transposon can be equipped with Gal4-VP16, resulting in sensitive detection of weak gene expression. Genes involved in a variety of cellular functions, from transcription to secretion, were mutated using our Gal4-based vector. The modified gene trap is highly mutagenic at molecular and phenotypic levels, resulting in isolation of two embryonic lethal and one post-embryonic lethal mutations which are revertible by Cre-mediated recombination.

## Results

### Gene trap vector design and features

**Figure 1:**
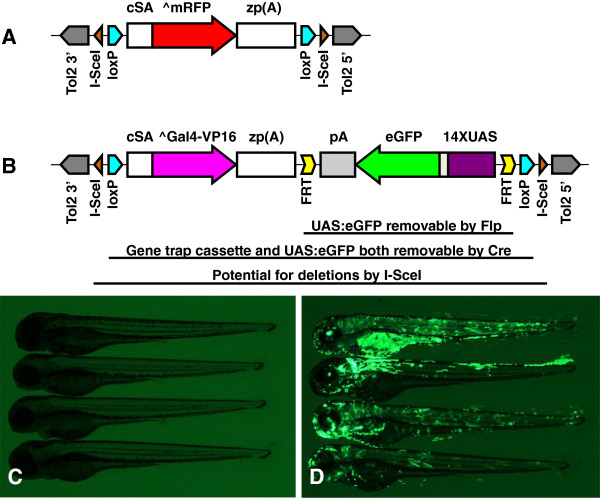
**Design of GBT-B1 trap. (A)** Parental vector GBT-R15 [27,28]. cSA denotes carp β-actin splice acceptor, ^mRFP denotes AUG-less mRFP, and zp(A) denotes zebrafish β-actin 3’ untranslated region and transcriptional termination / polyadenylation cassette. Tol2 5’ and Tol2 3’ are miniTol2 transposon arms as described in [37]. **(B)** Features of GBT-B1 trap. ^Gal4-VP16 denotes AUG-less Gal4-VP16, and the 14XUAS:eGFP cassette is from [34]. **(C)** Embryos injected with GBT-B1 without Tol2 transposase mRNA. **(D)** Embryos injected with GBT-B1 and Tol2 transposase mRNA.

Our GBT-B1 (for *G*ene *B*reaking *T*ransposon- *B*ipartite 1) vector is composed of several components that in concert ensure efficient mutagenesis, evaluation of the trapped gene’s expression profile and enable manipulation of resulting mutant alleles. It is based on the GBT-R15 vector (Figure 1A) [27,28], with AUG-less mRFP (^mRFP) replaced by AUG-less Gal4-VP16 (^Gal4-VP16) (Figure 1B). For direct detection of gene trap events, GBT-B1 vector also contains a UAS:eGFP cassette [23,34,36]. In addition, the vector has FRT, loxP and I-SceI meganuclease sites to facilitate manipulation of insertional alleles. The loxP and I-SceI sites, originally present in the parental GBT-R15 vector [27,28], flank Gal4-VP16 and UAS:eGFP sequences. The two loxP sites are in direct orientation and therefore permit excision of the gene trap leading to reversion of the mutations, as demonstrated for GBT-R15 mutations in *gabbr1b* and *tnnt2* genes [27,28].

In Drosophila, P-element integrations can be converted into deletions (deficiencies) by imprecise excision of the transposon [38,39] (reviewed in [40]). As Tol2 transposon does not appear to be prone to imprecise excision, we flanked our gene trap cassette with two inverted I-SceI meganuclease sites. While I-SceI is mainly used to facilitate transgenesis in zebrafish, it is used to study DNA repair pathways in other systems [41-44]. We anticipate that double strand breaks induced by I-SceI meganuclease may be repaired by error-prone repair mechanisms, leading to generation of local deletions large enough to remove one or more coding exons of the mutated gene.

To test if our gene trap vector containing Gal4-VP16, UAS:eGFP and additional features retained the specificity of GBR-R15, we injected this vector into 1-cell zebrafish embryos with and without Tol2 transposase mRNA. Without Tol2 transposase mRNA, there was no expression of eGFP (Figure 1C). When Tol2 transposase was included, almost all embryos had GFP-positive cells, with 20-30% expressing GFP quite highly and/or in tissues of different embryonic origin (Figure 1D). We concluded that GBT-B1 is unable to express eGFP unless integrated into the genome, and therefore may have sufficient fidelity to function as a gene trap.

Gal4-VP16 has been shown to be toxic when overexpressed, and an attenuated version of the activator, Gal4-FF, has been shown to activate expression of UAS-controlled transgenes in zebrafish [32,45-47]. We therefore constructed a second gene trap vector, named GBT-B2, which contains Gal4-FF in place of Gal4-VP16. When injected into zebrafish embryos, GBT-B2 produced significantly lower level of eGFP expression, suggesting that Gal4-FF is a significantly less potent transcriptional activator than Gal4-Vp16 (Additional file 1: Figure S1). We screened 58 F0 fish injected with GBT-B2 and failed to recover any gene traps. Gal4-FF may be too weak a transcriptional activator to function in the context of a highly stringent gene trap requiring a translational fusion with the N terminus of the protein encoded by the mutated gene.

### UAS:mRFP tester lines

**Figure 2:**
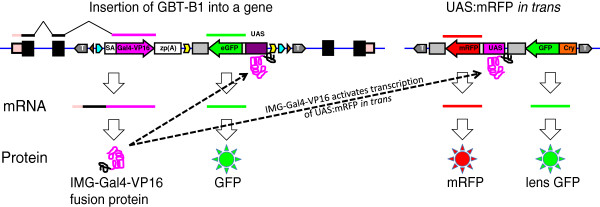
**Confirmation of GBT-B1 gene traps by transactivation of UAS:mRFP.** A gene trap event is depicted on the left, the UAS:mRFP tester transgene on the right. Solid black boxes and lines denote exons, pale pink boxes and lines denote untranslated regions of the insertionally mutated gene (IMG). Color scheme and shape used is identical to Figure 1. T denotes miniTol2 inverted repeats, UAS denotes 14 X Gal4 UAS, Cry denotes lens-specific γCry promoter from pT2/γCry:GFP cassette pDB387 in [19].

Integration of GBT-B1 into an intron of a protein-coding gene in sense orientation is expected to result in a fusion transcript between the 5’ end of the IMG (*I*nsertionally *M*utated *G*ene) mRNA and Gal4-VP16 (Figure 2). If the reading frames of the upstream exon of the IMG and Gal4-VP16 match, a fusion protein composed of the N-terminal portion of the protein encoded by IMG and Gal4-VP16 will be translated. For a gene trap to be detected, this fusion protein has to enter the nucleus, bind the 14XUAS and activate eGFP expression. However, eGFP expression may also result from an enhancer trap event if the minimal promoter in front of eGFP falls under the control of a nearby enhancer (Additional file 2: Figure S2, see also [48]). It is critical to distinguish between gene- and enhancer-trap events. Gene traps represent integrations into genes that disrupt their expression, while enhancer trap events are unlikely to be integrations into genes and consequently unlikely to result in any loss-of-function phenotypes. To distinguish between these two classes of events, we have generated two 14XUAS:mRFP transgenic lines. In case of a *bona fide* gene trap event, the IMG-Gal4-VP16 fusion protein will activate the expression of UAS-driven mRFP *in trans*. In case of an enhancer trap, eGFP is produced in the absence of Gal4-VP16 and UAS:mRFP will not be activated.

Our first gene trap tester line, *Tg(miniTol2/14XUAS:mRFP)*, was established based on weak leaky expression of mRFP in the nervous system. Leaky mRFP expression in the absence of transactivation complicated gene trap screening, and we no longer use this transgenic line. Our second UAS:mRFP tester line is *Tg(miniTol2/14XUAS:mRFP,* γ*Cry:GFP)tpl2* (*Tg(UAS:mRFP)tpl2* for brevity hereafter), marked by a lens-specific GFP cassette from [19]. To reduce the possibility of mixing up putative founder or F1 gene trap fish with *Tg(UAS:mRFP)tpl2* tester fish, we bred *Tg(UAS:mRFP)tpl2* onto a homozygous *brass* background and inject gene traps into fish with wild type pigmentation pattern.

### Screening strategies

**Figure 3:**
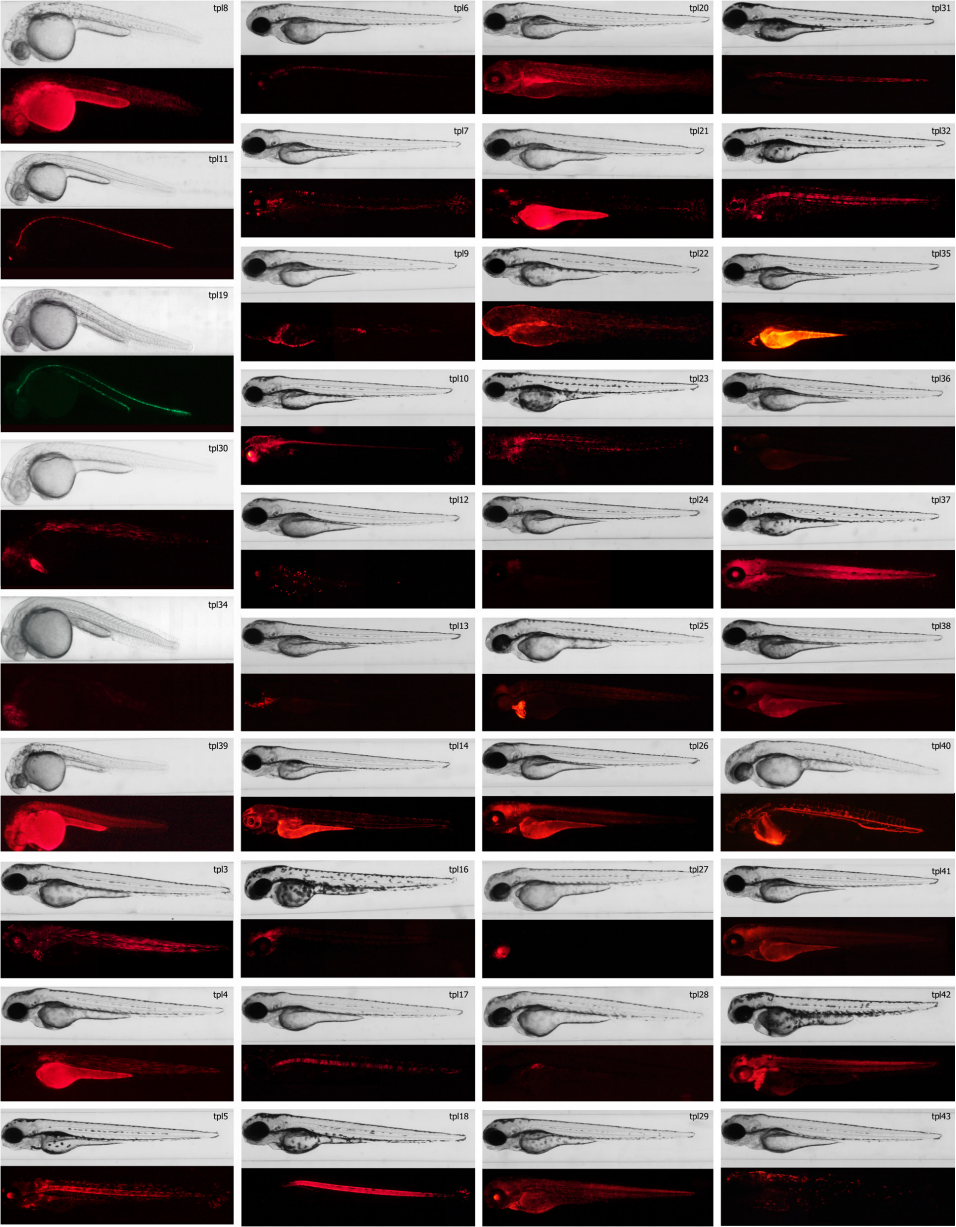
**Expression patterns recovered from the gene trap screen.** Each gene trap line is represented by two images: brightfield and fluorescence. Most of the lines are represented by expression pattern of the fluorescent reporter observed in 3dpf embryos, except for those lines were reporter’s expression pattern is best visible in embryos at earlier stages of development. The latter include lines *tpl8*, *tpl11*, *tpl19*, *tpl30*, *tpl34* and *tpl39* for which embryos were imaged at 1 dpf, as well as lines *tpl4*, *tpl6*, *tpl16*, *tpl21*, *tpl22*, *tpl25*, *tpl27*, *tpl28*, *tpl40* and *tpl42* that are represented by 2 dpf embryos.

The gene trap vector DNA was mixed with Tol2 transposase mRNA and injected into the yolk of 1-cell zebrafish embryos. Injected embryos were screened for GFP expression at 3 days post fertilization (dpf). Approximately 30% of embryos with the brightest GFP expression (Figure 1D) were selected and raised to establish an F0 pool for screening. The pilot gene trap screen was carried out in two stages. In the first stage, the F0 fish were incrossed, and all GFP-positive embryos were raised. GFP-positive fish were then crossed to *Tg(miniTol2/14XUAS:mRFP)* or *Tg(UAS:mRFP)tpl2* to distinguish between gene trap events (mRFP-positive) and enhancer trap events (mRFP-negative). Seventy fish were screened and 13 gene trap lines were established from this work. We have also discovered at least 10 enhancer trap events. Enhancer traps were discarded with the exception of *Et(GBT-B1)*^*tpl1*^, which displayed a highly specific vascular expression pattern [48]. In the second stage of the screen, GBT-B1-injected F0 fish were screened by crossing to the *Tg(UAS:mRFP)tpl2* line. We screened two hundred putative F0 fish and recovered 28 gene trap events from the second stage of the screen. Altogether, we screened 270 putative F0 fish and recovered 41 gene trap lines with diverse expression patterns (Figure 3).

### Identification of insertionally mutated genes (IMGs)

To estimate the number of integrations present in F2 fish, we have performed Southern hybridization analysis with an eGFP probe on a pool of 20 GFP positive and a pool of 20 GFP negative embryos from 13 different lines. The analysis revealed that the number of transposon insertions varied from 1 to 12 (data not shown) among different lines, and in most of them there was only a single integration linked to GFP.

Two complementary strategies were used to identify insertionally mutated genes: inverse PCR (iPCR) and 5’ RACE. iPCR is the preferred method for mapping IMGs as it permits identification of the exact genomic position of the gene trap integration. In contrast, 5’RACE only identifies the 5’ exon(s) of the trapped gene, but does not reveal the exact genomic position of gene trap integration, which makes it challenging to design genotyping primers.

iPCR analysis led to successful identification of IMGs in 21 of the lines while for the remaining 20 lines iPCR results were inconclusive, yielding either repetitive sequences that have multiple matches in the zebrafish genome, sequences that mapped to short contigs devoid of protein coding genes, or sequences that did not map onto current assembly of the zebrafish genome. In some cases, inverse PCR failed to produce any bands at all. For the majority of candidate IMGs (cIMGs) identified by iPCR, the presence of a fusion mRNA in GFP-positive but not GFP-negative embryos was also confirmed by conducting RT-PCR with a cIMG’s exonic primer upstream of the integration and a reverse Gal4 primer. Fusion mRNAs were then sequenced to verify continuity of the reading frame between the upstream IMG exon and Gal4-VP16.

To identify additional IMGs, we performed 5’-RACE on four gene trap lines that failed iPCR mapping: *tpl3*, *tpl8*, *tpl9* and *tpl15*. Similarly to iPCR, 5’ RACE was performed on RNA from batches of 20 GFP positive embryos following a published protocol [28]. Linkage of a given 5’ RACE product to GFP expression was confirmed by RT-PCR on mRNA from batches of 20 GFP-positive and GFP-negative embryos collected independently of the 5’RACE procedure. We successfully identified cIMGs in all four lines. For gene trap line *nudC*^*tpl3*^, exons upstream of the gene trap integration site are missing from Zv9 zebrafish genome assembly, which explains why inverse PCR failed to map this integration. In gene trap line *zfp36l1a*^*tpl8*^, the transposon integrated into a relatively short (4 kb) intron. We were able to map the exact position of the gene trap integration by carrying out PCR using exon primers in combination with Tol2 inverted repeat primers. We were unable to determine the exact position of the gene trap integration in lines *nfe2l1*^*tpl9*^ and *eef1a1l1*^*tpl15*^.

**Figure 4:**
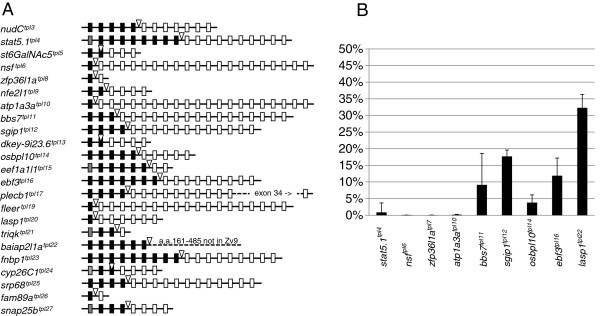
**Characterization of GBT-B1 gene trap events at the molecular level. (A)** Schematic illustration of molecularly characterized gene trap events. Gene trap integration is indicated by an open triangle. Exons upstream of gene trap integration are shown as black (coding exons) or grey (non-coding exons) boxes. Exons downstream of gene trap integration are shown as open boxes. Integrations into *jam3b* and *fam46bb* are not shown because they do not form in-frame fusions with the upstream exon. **(B)** Levels of wild-type transcript present in homozygous gene trap mutants.

Altogether, we established 41 gene trap lines and successfully identified IMGs linked to GFP expression in 25 of them (Table 1, Figure 4A). In 20 cases, gene trap integration has occurred into an intron following an exon ending in phase 0 with respect to the gene’s reading frame. This is the expected scenario, as the reading frame of Gal4-VP16 in our gene trap vector is in phase 0 with respect to the splice site. Among the five cases not conforming to this expected scenario, four are integrations into exons. In gene trap lines *st6GalNAc5*^*tpl5*^, *dkey-9i23.6*^*tpl13*^ and *cyp26C1*^*tpl24*^, the transposon integration occurred into exons immediately following an exon in phase 0. We did not perform RT-PCR on *dkey-9i23.6*^*tpl13*^, but in gene trap lines *st6GalNAc5*^*tpl5*^ and *cyp26C1*^*tpl24*^ RT-PCR yielded fusion transcripts consisting of the upstream exon and Gal4-VP16. This occurs if splicing machinery skips the endogenous splice acceptor upstream of the exon into which the gene trap integrated and used the gene trap splice acceptor instead. The fourth gene trap line with an exonic gene trap integration linked to GFP expression is *fam46bb*^*tpl18*^. In this case, the gene trap has integrated into the first exon of the gene, downstream of the ATG but 14 base pairs upstream of exon/intron boundary. It is likely that another linked gene trap integration we failed to identify may be responsible for the GFP expression pattern. Nonetheless, *fam46bb*^*tpl18*^ can be considered a null mutant of *fam46bb*. The fifth non-canonical case of gene trap integration is presented by *jam3b*^*tpl7*^ line. In this case, gene trap integration into the first intron of *jam3b* gene is linked to GFP expression, and expression in our gene trap line closely resembles the published expression pattern of *jam3b* (see zfin.org). However, the first exon of *jam3b* ends in phase 1, which should prevent translation of Jam3b-Gal4VP16 fusion protein. We confirmed this out-of-frame fusion transcript by RT-PCR. We also observed that two gene trap integrations were linked to GFP expression by Southern hybridization (data not shown), indicating that additional integrations on chromosome 21 may be responsible for the gene trap expression pattern.

**Table 1:** Molecularly identified GBT-B1 gene trap lines

	**Linked trap integration**	**Zv9 integration**	**IMG**	**IMG-linker-^Gal4-VP16**
tpl3		19:30.5 Mb	*nudC*	YRWTQSLSEVDFSRNSPGYQK
tpl4	CTG*g*tcagtctttgaagaggatcaggcgttcagacagacggggggcagaatctgttacagaag	3:16936744	*stat5.1*	GTLSAHFRNMDFSRNSPGYQK
tpl5	CTGtttctcgccatcaccatgtgcaccagtctgttgttcgtgtataacgtcagctacaataat	2:9043594	*st6GalNAc5*	QGYSSIIEHKDFSRNSPGYQK
tpl6	CTGcttactagtactggaatggacccgcttatgccttcagaactgccttaatccttcgtgaca	3:34884583	*nsfa*	MATRDFSRNSPGYQK
tpl7	CTGagtgactataaatgcctgaaaaatcaactcatgtaagcacaaaatccttttgaacttttg	21:24267477	*jam3b*	not in frame
tpl8	CTGcagctggatcagtttgtgttgtggaatgttgaggcaggctttctttctaggattaaatgc	17:37963171	*zfp36l1a*	FDFSEMINNKDFSRNSPGYQK
tpl9		12:30.4 Mb	*nfe2l1*	QDIMSIMELQDFSRNSPGYQK
tpl10	CTGgggtgtctgctgattcaccaagctccaaaatattgaattcctttcgtcattaaatggcct	19:7006530	*atp1a3a*	MEDFSRNSPGYQK
tpl11	CTGtgaggctgtgtgaattttagcctcagtttcctgttctttgctgacagaagtggcacctgg	14:49326467	*bbs7*	CFGMKKGEAVDFSRNSPGYQK
tpl12	CTGtttaattggtgttttttatttaatagattataacagttcgggtctgacattctcatgctg	6:30448559	*sgip1*	GNIALSPSPLDFSRNSPGYQK
tpl13	CTGggcacatttaatgtgtctgagtcaatgccgagtgaggcaagtcaaggtcagcgaagtgat	1:45249862	*dkey-9i23.6*	MEEQTAKDFSRNSPGYQK
tpl14	CTGaaaattcaacaagatatgtttgattctcaatttgaagtctctgacttttgattgcagtct	16:42014825	*osbpl10*	SSSSVSWAVCDFSRNSPGYQK
tpl15		19:45.3 Mb	*eef1a1l1*	PMEAANFTSQDFSRNSPGYQK
tpl16	CTGgatagaacagggtttttccccccccccacaaaccataatcgcagacaattccaacccaaa	12:44187402	*ebf3*	GNPRDMRRFQDFSRNSPGYQK
tpl17	CTGttagtgtatatacagtgctcggcataactggctacaacccattttgaaaatgaatatttt	16:6676846	*plecb1*	LLEVLSGETLDFSRNSPGYQK
tpl18	CTGggcatatcgtacaggtaaagttacaggacccatatataaaatgaaaatcagttttaaaaa	19:14006402	*fam46bb*	Into exon 1
tpl19	CTGacaagctaacaggctaaccttaatttagttaacgtttcatcttctctcatctgctgtttg	3:28982811	*fleer*	GEYTATVYKMDFSRNSPGYQK
tpl20	CTGtggtcaacccctcaaacaaccgccaaccccactcaccccctaggaggagaataaaaacta	3:15727276	*lasp1*	PTEKVNCLDKDFSRNSPGYQK
tpl21	CTGcctgagagcaagtcaagcagtctccatattgatgaggcagagaagagctgtttgactctc	19:34858311	*triqk*	EAKRSAPGIRDFSRNSPGYQK
tpl22	CTGacatgaccagcaatttactgcagctgccattggttgtgaaaagaattagtggcatgtgcg	12:18869291	*baiap2l1a*	SSKYEIKENEDFSRNSPGYQK
tpl23	CTGagggattcatatttgttacatttgtaaaggcgattagttgtctttaaaaagtgagtaaaa	21:6304212	*fnbp1*	TLWPFIKKNKDFSRNSPGYQK
tpl24	CTGgctgtagagatattaataaatatgttcaacttacttttcttttcctcttgtgcagatctc	17:19362393	*cyp26c1*	VGETFHWLFQDFSRNSPGYQK
tpl25	CTGagtaattaaactttgtccattgatttaataaaaaagctaattttaatactaagcaggggt	6:21614143	*srp68*	VDAKTKLEAQDFSRNSPGYQK
tpl26	CTGcacatacaataatatgctatttagatttgatacgtttttgttaaatagtaatattgttaa	20:4058970	*fam89A*	AALALLRKEMDFSRNSPGYQK
tpl27	CTGctggggcgatagatagactttccagttagcactatctaatgcgatcccgtgaacagcatt	17:12186310	*snap25b*	TRRMLQLVEEDFSRNSPGYQK

First column, gene trap line. Second column, genomic sequence adjacent to 3’ end of the gene trap integration. The capital CTG are the last three nucleotides of Tol2. Third column, location of the gene trap integration on Zv9 zebrafish genome assembly. Fourth column, insertionally mutated gene. Fifth column, sequence of the IMG-Gal4-VP16 fusion protein, IMG sequence is highlighted in aqua, Gal4 sequence is highlighted in magenta. The “linker” sequence is encoded by the linker between splice acceptor and Gal4 in GBT-B1.

### Correspondence between expression patterns of insertionally mutated genes and fluorescent reporters

Our gene trap screen yielded a variety of expression patterns, ranging from fairly ubiquitous to tissue-specific (Figure 3). Expression patterns of UAS:eGFP in *cis* and UAS:mRFP in *trans* matched very closely. Consistently with published observations [49], eGFP expression was quicker to appear in all gene trap lines, but mRFP expression was more robust in later stages of development. Expression patterns of many of the mutated genes have been previously described by others, and data has been deposited to Zebrafish Information Network (zfin.org), including not spatially restricted expression patterns noted for *nudc* (*tpl3*), *stat5.1* (*tpl4*), *eef1a1l1* (*tpl15*) and *lasp1* (*tpl20*). Other expression patterns available through ZFIN reasonably closely match our observed expression patterns with some notable exceptions. Several of the gene traps display pronounced mRFP fluorescence in the yolk (*tpl4*, *tpl9*, *tpl14*, *tpl21*, *tpl26*, *tpl35*, *tpl39*), while gene expression in the yolk is not typically observed by *in situ* hybridization. This may be non-specific, or may reflect accumulation of fluorescent protein from maternal contribution or from earlier gene expression in yolk syncytial layer. A separate subset of gene traps displays mRFP expression in the notochord (*tpl5*, *tpl17*, *tpl18*, *tpl31*, *tpl32*) at 3 dpf. Similarly to yolk, notochord is not a prominent expression domain when gene expression at 3 dpf is analyzed by *in situ* hybridization (zfin.org and [50]). mRFP expression in the notochord may be a remnant of earlier expression, or may reflect non-specific background. A particularly instructive example is presented by the *baiap2l1a*^*tpl22*^ gene trap. By *in situ* hybridization, *baiap2l1a* is expressed in the periderm and notochord before but not after the 25-somite stage. In 2-day embryo, *baiap2l1a* is expressed in the pronephric duct, in the general area of the pharynx and in the brain (zfin.org and [50]). In contrast, mRFP expression in the skin and the notochord is observed through 3 dpf in *baiap2l1a*^*tpl22*^ gene trap line, in addition to highly pronounced expression in the areas of kidney tubules, pharynx and lower jaw (Figure 3). It has been noted that turnover of mRFP *in vivo* is very slow [49], which may explain the presence of UAS-driven mRFP but not *baiap2l1a* mRNA in the skin and the notochord at 3 dpf.

In the *nsfa*^*tpl6*^ gene trap line, we observed expression of GFP throughout the developing nervous system in a pattern largely consistent to the previously reported expression of *nsfa*[51,52]. However, the gene trap line *nsfa*^*tpl6*^ also expresses GFP and RFP in the pancreas (Figure 3). To investigate if this pancreatic fluorescent protein expression corresponds to endogenous expression of *nsfa*, we performed whole-mount *in situ* hybridization using a DIG-labeled riboprobe antisense to *nsfa*. Robust *in situ* hybridization signal in the nervous system was detectable after a short staining period. Longer incubation yielded a clear hybridization signal in pancreas as well (Additional file 3: Figure S3). This indicates that endogenous *nsfa* transcript is present in the pancreas. Relatively low abundance of *nsfa* transcript in the pancreas compared to the nervous system explains why it was missed by previous studies in zebrafish. Importantly, *nsf* is known to be expressed in human pancreatic beta-cells [53].

**Figure 5:**
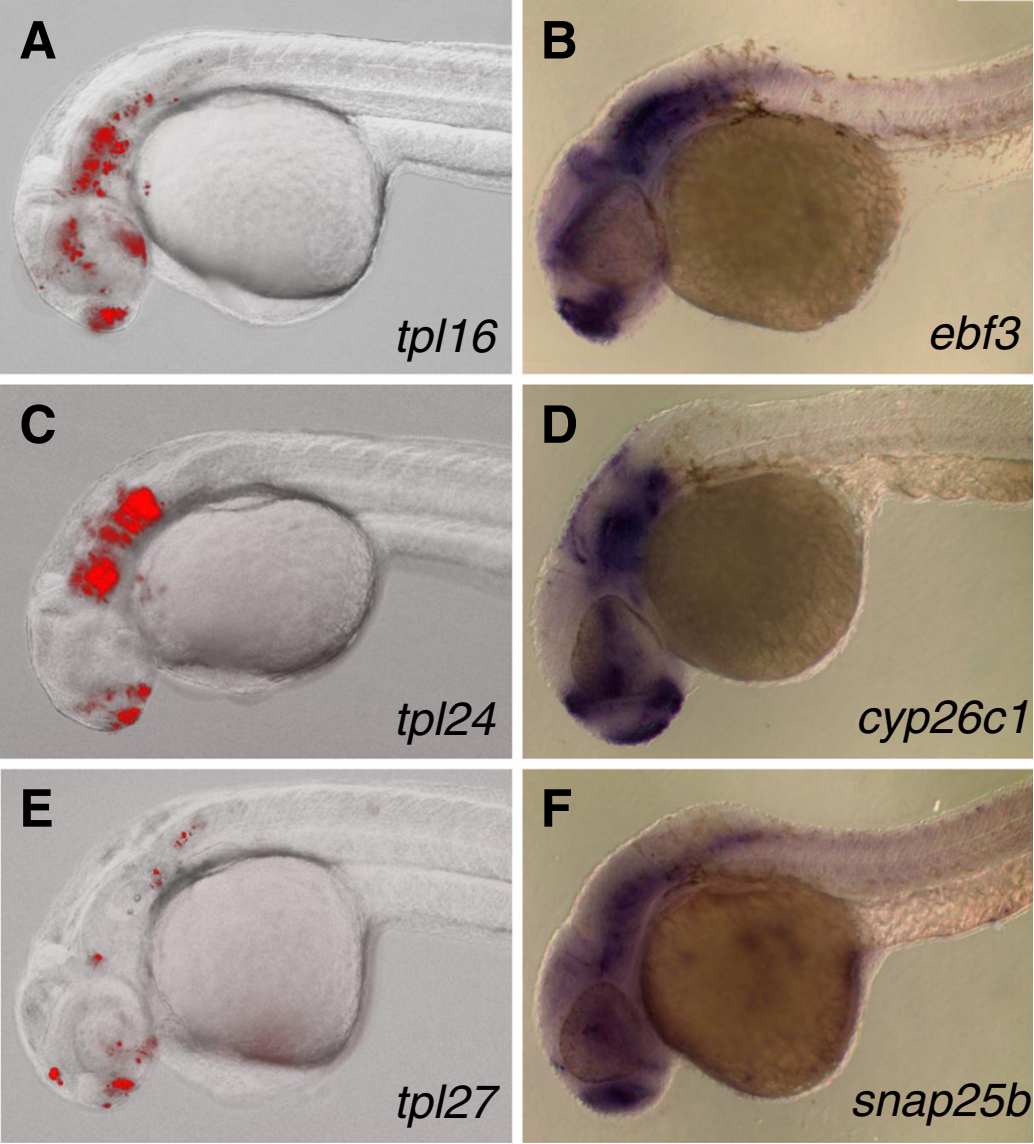
**Comparison between patterns of UAS-driven mRFP fluorescence and endogenous gene expression by whole mount *in situ *hybridization.** Embryos containing *ebf3*^*tpl16*^, *cyp26c1*^*tpl24*^ and *snap25b*^*tpl27*^ gene traps and *Tg(UAS:mRFP)tpl2* reporter where photographed on Zeiss AxioImager microscope at 30–32 hpf **(A**, **C**, and **E** respectively**)**. Expression of endogenous genes detected by whole mount *in situ* hybridization using antisense probes against *ebf3***(B)**, *cyp26c1***(D)** and *snap25b***(F)** on 30 hpf embryos.

While all of our Gal4 gene traps can be used as drivers to express other transgenes in tissues expressing mRFP (Figure 3), lines displaying restricted neuronal expression are of particular interest given presence of well-established Gal4/UAS based tools to modulate and detect neuronal activity [32,35,54-57]. We therefore decided to test how closely expression of mRFP *in trans* corresponds to the endogenous expression of three genes expressed in overlapping but different neuronal domains: *ebf3*, *cyp26c1* and *snap25b* (Figure 5). We found that expression of mRFP largely corresponds to the expression of endogenous gene, but in an incomplete and/or mosaic pattern. Incompleteness of mRFP expression compared to the endogenous expression may in part be due to the delay in mRFP fluorescence because of slow maturation of mRFP as well as the additional step of transcriptional activation by Gal4-VP16. Mosaicism of mRFP expression compared to the expression of endogenous gene is most likely due to partial silencing of Gal4 UAS. Thus, while BGT-B1 gene traps can be used as highly specific Gal4 drivers, additional steps should be taken to ensure that the transgene of interest is indeed expressed in the cells that are being targeted.

### Assessment of mutagenicity at the molecular level

One of the key questions regarding any mutagenesis strategy is the ability to generate complete loss-of-function (null) alleles. Reverse genetic strategies such as tilling, zinc finger nucleases and TALEN nucleases in zebrafish and homologous recombination in the mouse are designed to ensure physical disruption of exons or splice sites, which greatly increases the probability of complete loss-of-function alleles. Generation of loss-of-function alleles by GBTs relies on the efficiency of the vectors splice acceptor and transcriptional termination signals in a given genomic context. To assess the mutagenic efficiency of our system, we have performed qRT-PCR to quantify the levels of wild type read-through transcript in 9 lines (Figure 4B). Expression levels were compared between three or four homozygous mutant embryos and three wild type siblings at 5 dpf. In four out of six lines, the amount of wild type transcript was below 1%, while one additional line had wild type transcript at almost 4%. In the remaining four lines, the amount of wild type transcript ranged from 9.5% to 32%.

It should be noted that lines harboring integrations into exons - *st6GalNAc5*^*tpl5*^, *dkey-9i23.6*^*tpl13*^, *fam46bb*^*tpl18*^ and *cyp26C1*^*tpl24*^ - were excluded from this qRT-PCR analysis because wild type transcript cannot be produced in these lines. We also excluded the gene trap line *flr*^*tpl19*^, because it had a severe embryonic phenotype suggestive of a null phenotype (see below). It is difficult to unequivocally interpret levels of tissue-specific specific transcript between wild type embryos and embryos with severe developmental defects.

### Assessment of mutagenicity at the phenotypic level

To test for overt homozygous phenotypes, we have in-crossed F2 or later generation heterozygous siblings from all gene trap mutant lines. Embryos were checked for overt phenotypes at 1 dpf, 3 dpf, 5 dpf. Two of our gene trap lines are insertional alleles of genes with previously characterized chemically-induced mutations: *nsfa*^*tpl6*^ and *flr*^*tpl10*^. All incrosses of *nsfa*^*tpl6*^ heterozygotes resulted in approximately 25% of embryos displaying failure to inflate the swim bladder and a progressive paralysis phenotype consistent with the previously published phenotype of *nsfa*^*st25*^*and nsfa*^*st53*^[51] (Additional file 4: Figure S4A and Additional file 5: Movie 1). PCR genotyping confirmed that all paralyzed embryos with non-inflated swim bladders were homozygous for the *nsfa*^*tpl6*^ gene trap allele (data not shown). Similarly, all incrosses of *flr*^*tpl10*^ heterozygotes resulted in 25% of embryos displaying abnormal body curvature and kidney defects similar to the phenotype of *flr*^*m477*^ homozygous mutants [58] (Additional file 4: Figure S4B and Additional file 6: Movie 2).

To test for postembryonic lethality, we raised GFP-positive fish from incrosses of 8 additional gene trap lines: *nudC*^*tpl3*^, *stat5.1*^*tpl4*^, *st6GalNAc5*^*tpl5*^, *zfp36l1a*^*tpl8*^, *nfe2I1*^*tpl9*^, *atp1a3a*^*tpl10*^, *bbs7*^*tpl11*^ and *sgip1*^*tpl12*^. For gene traps that do not affect survival of homozygotes, we expected 1/3 of adult fish to be homozygous and 2/3 fish to be heterozygous for the gene trap. In all but one line (*atp1a3a*^*tpl10*^) we observed the expected Mendelian ratio of homozygous mutants versus heterozygous fish. In the *atp1a3a*^*tpl10*^ gene trap line, no homozygous mutants were identified after genotyping the initial clutch of 14 GFP-positive adult survivors, even though homozygous embryos did not display overt phenotypes at 5 dpf (Additional file 7: Figure S5). We then followed the survival rate among 86 GFP positive embryos from 4 different clutches of heterozygous in-cross over a period of one month. We discovered that a severe drop in survival occurred between 8 dpf and 10 dpf with no further change beyond 20 dpf. Forty five percent (42/86) of larvae survived to one month. We then genotyped adult fish (n = 24) from two additional clutches and failed to find homozygous mutants among them. Together with the initial genotyping data we obtained 0/38 homozygous embryos instead of the expected ratio of approximately 13/38 (p < 0.001). This phenotype is consistent with postembryonic lethality observed in *atp1a3* mutant mice [59].

Morpholino (MO) knockdown phenotypes have been published for two mutants recovered in our screen, *atp1a3a*^*tpl10*^ and *bbs7*^*tpl11*^[60,61]. Morpholino knockdown of Atp1a3a resulted in dilated brain ventricles and electrophysiological defects in Rohon-Beard neurons, resulting in defective touch response [61]. In incrosses of *atp1a3a*^*tpl10*^ heterozygotes we did not observe embryos with severely dilated (or otherwise morphologically defective) brain ventricles, but we have not assessed them for more subtle defects (Additional file 7: Figure S5A). We also did not observe severe touch response defects reported in morpholino injected embryos at 60 hpf. However, larval lethality of *atp1a3a*^*tpl10*^ homozygotes clearly supports an essential role for Atp1a3a in neural development and/or physiology.

For *bbs7*, Yen and colleagues observed absent or reduced Kupffer’s vesicle (KV) in 28.1% of embryos injected with 500 μM solution of morpholino. The percentage of embryos with KV defects was reduced to 6.7% when 250 μM solution of the same morpholino was used, indicating that this phenotype was highly dose-dependent. We did not observe a significant fraction of embryos with KV defects in *bbs7*^*tpl11*^ heterozygous incrosses (data not shown). Yen and colleagues also noted that approximately 14% of embryos injected with either 250 μM or 500 μM solution of *bbs7* morpholino displayed defects in cardiac jogging: the first morphologically observable indication of left-right patterning [60]. In our incrosses of *bbs7*^*tpl11*^, we observed a low percentage (<5%) of embryos with heart looping or jogging defects or delay at 1 dpf, but a majority of these embryos had normal hearts by 3 dpf. Notably, embryos homozygous for *bbs7*^*tpl11*^ retained over 9% of full-length transcript, and the level of endogenous full-length *bbs7* transcript varied 14-fold among homozygous embryos while the variation was only 2-fold among wild type controls. Furthermore, *bbs7*^*tpl11*^ homozygotes are viable and fertile. Based on these observations, we believe that *bbs7*^*tpl11*^ is a hypomorphic allele. Hypomorphic nature of *bbs7*^*tpl11*^ allele explains why we did not observe phenotypes nearly as severe as noted for high-dose morpholino knockdown. It has also been suggested that mutations in modifier genes are required for full penetrance of Bardet-Biedl Syndrome [62-65], making it possible that the observed *bbs7* MO phenotypes may be specific to the genetic background in which they were observed.

### Reversion of gene trap mutations by Cre recombinase

The internal components of the GBT-B1 vector (the gene trap cassette and the UAS:eGFP cassette) are flanked by loxP sites in direct orientation, analogously to the arrangement in GBT-R15 and GBT-RP2 [27,28]. Since mutagenic properties of gene breaking transposons are brought about by the splice acceptor and polyadenylation/transcriptional termination signals, removal of these components should lead to reversion of the insertional mutation.

**Figure 6:**
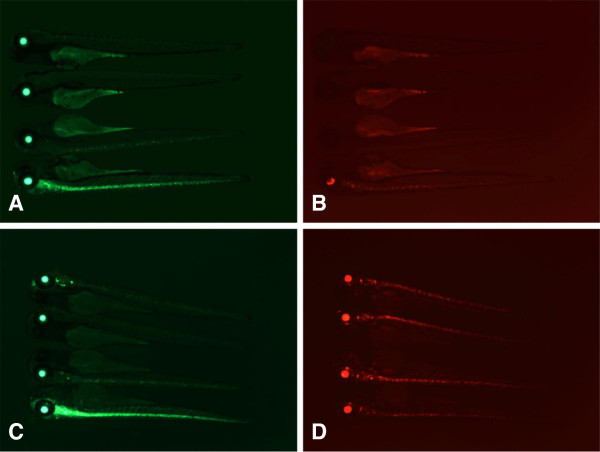
**Manipulation of the *nsfa***^***tpl6 ***^**gene trap using Cre and Flp recombinases.** Embryos containing the *nsfa*^*tpl6*^ gene trap and *UAS:mRFP*^*tpl2*^ were injected with 25 pg of Cre **(A, B)** or 600 pg of eFlp **(C, D)** mRNA. Groups of four representative embryos, with the bottom embryo representing low recombinase activity. Note simultaneous loss of GFP and RFP in panels **A** and **B** (injection of Cre RNA), and loss of GFP without loss of RFP in panels **C** and **D** (injection of eFlp RNA).

To test the efficacy of gene trap excision by Cre recombinase, we crossed *nsfa*^*tpl6*^ to *Tg(UAS:mRFP)tpl2* homozygotes and injected 25 pg of *in vitro* transcribed Cre mRNA into the yolks of 1-cell embryos. We found that Cre recombinase was extremely efficient at excising the gene trap cassette, as evidenced by mosaic and nearly complete loss of both mRFP and eGFP expression in Cre-injected embryos (Figure 6A, B). We then tested if this Cre activity is sufficient to revert mutant phenotypes in injected embryos. We injected 75 pg of *in vitro* transcribed Cre mRNA into the yolks of 1-cell embryos obtained from incross of gene trap (*nsfa*^*tpl6*^, *flr*^*tpl10*^ and *atp1a3a*^*tpl10*^) heterozygotes. For *nsfa*^*tpl6*^ and *flr*^*tpl10*^, we then prepared DNA from individual non-paralyzed 5 dpf embryos with inflated swim bladders and performed PCR genotyping for gene trap homozygocity. In both cases, we found 5/24 genotyped embryos to be homozygous for the gene trap (data not shown).

For *atp1a3a*^*tpl10*^, we scored Cre-injected embryos for GFP and raised GFP-positive embryos to adulthood. Adult fish were genotyped, and 3/10 were found to be homozygous for the reverted *atp1a3a*^*tpl10R*^ allele (data not shown), demonstrating efficient reversion of the mutant allele.

### Removal of UAS:eGFP by Flp recombinase

Our gene trapping cassette has a built-in UAS:eGFP component, which allows instant visualization of the trapped gene’s expression pattern. To increase versatility of our mutant lines, we have flanked UAS:eGFP sequences by direct FRT sites. Upon expression of Flp recombinase, recombination between the FRT sites would result in excision of the UAS:eGFP reporter without affecting the mutagenic Gal4-VP16 component. Excision of the eGFP reporter would make the GFP channel available for utilization in other GFP-reliant transgenic applications. For the proof-of-principle of Flp-recombinase functionality, we injected 600 pg of in vitro transcribed Flp recombinase RNA into the yolks of 1-cell embryos heterozygous for the *nsfa*^*tpl6*^ gene trap and containing *Tg(UAS:mRFP)tpl2*. As expected, injection of Flp mRNA did not lead to reduction of mRFP expression. Somewhat surprisingly and in contrast to injection of Cre mRNA, a majority of the embryos did not display a significant reduction in eGFP expression either. Only about 10-15% of the embryos displayed significant loss of eGFP (Figure 6C, D). Embryos with significantly reduced GFP expression were raised to adulthood and six resulting adults were outcrossed to *Tg(UAS:mRFP)tpl2*. Germline-transmited Flp-mediated UAS:eGFP excision events were scored by complete absence of eGFP expression in the presence of mRFP. Five out of six outcrossed adults displayed complete loss of eGFP expression in the germline (Additional file 8: Table S2). As expected, the Flp-deleted allele of *nsfa*^*tpl6*^ retains the ability to activate *Tg(UAS:mRFP)tpl2 in trans,* and 50% of embryos display mRFP expression. We conclude that UAS:eGFP cassette can be readily removed from GBT-B1 gene trap lines.

## Discussion and conclusions

The main principle underlying our insertional mutagenesis system is shared with the recently reported 5’ gene trap vectors GBT-R14, R15 and R16 [27,28], which use highly efficient splice acceptor and polyA to disrupt endogenous transcripts and mRFP to visualize the mutated gene’s expression pattern. We have expanded the versatility of these gene-breaking transposons by splitting the expression-reporting core into two separate entities. We have replaced mRFP with Gal4-VP16 in the mutagenic entity of the vector. To report the presence of Gal4-VP16 we included a Gal4 UAS reporter (UAS:eGFP). The UAS:eGFP component is removable by Flp recombinase for situations where the GFP channel is needed for other purposes such as visualization of another transgene. Replacement of mRFP with Gal4-VP16 serves a dual purpose. First, transcriptional activator property of Gal4-VP16 amplifies the signal of the trapped gene, enabling visualization of low level IMG expression. For example, Gal4-VP16 dependent GFP and RFP expression was observed in the pancreas of the *nsfa*^*tpl6*^ gene trap line – an organ which was previously not known to express *nsfa*. Second, while mutant lines with no phenotype have rather limited research value, our “non-phenotypic” gene trap lines can be used as Gal4 drivers for ectopic expression of any UAS-controlled transgene of interest. Notably, neither epigenetic silencing of UAS:eGFP nor removal of UAS:eGFP by Flp affect the ability of our gene traps to act as Gal4 drivers. Furthermore, the standard considerations apply when using our gene traps as Gal4 drivers: the UAS:driven transgene may be affected by silencing in some or all tissues, and it may or may not express in any individual cell, as illustrated for mRFP in Figure 4. In that sense, our gene traps are not different from previously published Gal4 enhancer- and enhancer/gene-traps [23,31,32]. We would like to note that compared to enhancer traps with *hsp70* minimal promoter, our gene trap does not exhibit background expression in non-specific tissues such as the heart and the muscle.

The use of Gal4-VP16 as the primary gene trap reporter may also raise some concerns. It has been noted that Gal4-VP16 can be toxic when expressed at high levels [32,45-47]. We recovered several lines with very high levels of fluorescent reporter (GFP and RFP) expression, for example *tlp26*, *tpl37* and *tpl39*, and did not observe overt phenotypes in incrosses of these lines. Furthermore, there is a significant difference in how Gal4-VP16 is expressed between different experiments. Our gene traps produce fusion protein under the control of a single-copy endogenous promoter, while other experiments used strong exogenous promoter constructs, potentially in multiple copies. In our gene trap lines Gal4-VP16 is expressed as a fusion protein with the N terminus of the protein encoded by the endogenous gene, which is not usually the case in other experimental paradigms. Together with the observation that Gal4-FF is inactive in our gene trap context, our data argues that the strength of Gal4-VP16 is just right for our gene trap context, and that expression levels our gene traps achieve are unlikely to cause of toxicity.

Another potential concern for using Gal4-VP16 as the primary gene trap reporter is that expression of UAS-controlled transgenes are susceptible to variegation and silencing in zebrafish [47,66]. To circumvent this problem, we employ a second reporter, UAS:mRFP, to ascertain the presence of the gene trap allele. We also tend to select high expressors when we propagate our gene trap lines. Nonetheless, after F5 generation, we no longer observe GFP expression in several lines including *ebf3*^*tpl16*^ and *fnbp1*^*tpl24*^. Despite of loss of GFP expression, these gene trap lines can still be used as Gal4 drivers, as they successfully transactivate UAS:mRFP. Replacing the 14XUAS with a less repetitive variant such as nrUAS [66] may be worthwhile if sensitivity for low expression levels can be retained.

The third potential concern for using Gal4-VP16 as the reporter may be the requirement that IMG-Gal4-VP16 fusion proteins must enter the nucleus, bind the DNA and activate transcription. This excludes a significant subset of genes as potential targets. For example, Gal4-VP16 fusions with proteins containing amino-terminal signal peptide would be unable to enter the nucleus. Such proteins constitute about a fifth of vertebrate proteomes [67]. Many other protein domains have been noted to affect protein localization within the cell. We were concerned that use of Gal4-VP16 as the primary reporter will introduce significant bias in intracellular functions of trapped genes, such that we would mainly target transcription factors and other proteins which primarily function in the nucleus. While the subset of trapped genes presented here is too small to exclude or confirm the possibility of such bias, we note that among the trapped genes, two are components of the SNARE complex involved in secretory pathway (*nsfa* and *snap25*), and two are involved in cilia biogenesis (*bbs7* and *fleer*). Thus, genes involved in a variety of cellular processes can be mutated using our vector. Furthermore, GBT-R15 and GBT-RP2 are especially suitable for trapping genes coding for secreted proteins [28], making our Gal4-VP16-based approach complementary.

It would be possible to expand the repertoire of genes amenable to trapping using Gal4-VP16 (e.g. eliminate bias against proteins with N-terminal signal sequence) by using viral P2A/T2A co-translation systems demonstrated to work in zebrafish [68,69]. We do not favor this strategy, since there appears to be a positive side effect of using Gal4-VP16 as the primary gene trap reporter. Among gene trap lines we characterized, six have integrations into intron 1, and two additional lines have integrations into exon 2, which effectively results in fusion transcript with exon 1 (Figure 4A). Gene traps using fluorescent reporters do not appear to have a similar 5’ bias [22,27,28,70,71]. The 5’ bias of our vector is likely brought about by the requirement that IMG-Gal4-VP16 fusion protein must enter the nucleus, bind DNA and activate transcription of eGFP and mRFP under the control of Gal4 UAS. One or more of these functions may be compromised by addition of a large polypeptide with one or more functional domains. As a consequence of this 5’ gene trap bias, a shorter part of the endogenous gene is expressed, which increases the likelihood of null alleles. Inclusion of P2A/T2A would eliminate this beneficial bias of Gal4-VP16 gene traps toward 5’ ends of genes.

Efficient mutagenic potential of our vector is observed at both molecular and phenotypic level. Among the 25 molecularly characterized lines, four are integration into exons and therefore null alleles. Almost half the lines analyzed by RT-PCR (4/9) display wild type transcript levels below one percent and therefore should be considered null alleles as well. A fifth has wild type transcript level below five percent, making it a possible null mutant, too. The *flr*^*tpl10*^ line was not analyzed by RT-PCR due to developmental abnormalities but displays a homozygous phenotype very similar or identical to that of the corresponding chemically-induced null mutant. Thus, it is safe to assume that well over 50% of mutants recovered with GBT-B1 will be null alleles. How does it compare to other published insertional mutagenesis systems? Assessment of mutagenicity of the only insertional mutant used in large scale in zebrafish so far, the pseudotyped retrovirus, demonstrated that 4/10 integrations into intron 1 resulted in transcript levels below 5%, while 1/8 integrations immediately upstream transcription start site and 0/5 integrations into other introns produced likely null alleles [72]. A more appropriate comparison would be with other transposon-based insertional mutagenesis systems. Unfortunately, mutagenicity of most other transposon systems was not systematically assessed at the molecular level [22,32,71]. Recently published Flex-based vectors reduced the levels of endogenous transcript to just below 7% [70], which is easily surpassed by our vector. It is also interesting to note that GBT-B1 appears to be more mutagenic than the parental vector GBT-R15 [27,28]. This may be caused by the addition of the UAS:eGFP cassette. Even though this cassette is in antisense orientation in the trap, the SV40 p(A) used to terminate transcription of eGFP is bidirectional [73]. Even though SV40 p(A) is not efficient enough to cause mutations on its own [26], it may contribute to reduction in endogenous transcript. Furthermore, several potential splice acceptor sites can be identified in the antisense strand of 14XUAS:eGFP cassette (http://wangcomputing.com/assp/index.html[74]). It is also possible that replacement of ^mRFP with ^Gal4-VP16 added an exonic splice enhancer or removed an exonic splicing silencer [25], thus improving the efficiency of the short carp β-actin splice acceptor used in both vectors. Each of these factors may account for the minor increase in mutagenicity of GBT-B1 compared to GBT-R15. However, mutagenicity of GBT-B1 is clearly lower than that of GBT-RP2 [28]. GBT-RP2 contains an additional carp β-actin splice acceptor in the 3’ gene trap component of the vector, which may contribute to higher mutagenicity of this vector. GBT-RP2 vector also uses a poly (A) which is derived from ocean pout antifreeze gene and is thought to contain a potential boundary element [75]. It would be interesting to test if replacement of zebrafish β-actin p(A) with ocean pout antifreeze p(A) would increase the mutagenicity of our vector to RP2 knockdown levels, or if an additional splice site would still be required.

While qPCR provides the precise degree of gene inactivation at the molecular level, the level of disruption needed to achieve a loss-of-function phenotype is likely to be different from gene to gene. Loss-of-function chemically-induced mutants have been previously described for two of the genes mutated in our screen, *nsfa*^*tpl6*^, *flr*^*tpl10*^. In both cases, the phenotypes of our insertional mutants appear to be indistinguishable from corresponding chemically-induced alleles [51,58]. Interestingly, gene trap integrations in these two genes have occurred into the first intron. Only 4/744 and 18/651 amino acids are retained by Nsfa- and Fleer-Gal4-VP16 fusion proteins, respectively.

Our mutagenesis system offers an ability to conditionally revert mutagenic insertions into non-mutagenic ones by Cre-recombinase mediated excision of the Gal-VP16 (and UAS:eGFP) sequences. Reversion of mutations by injection of Cre mRNA is very useful in determining causal relationship between gene trap integrations and observed phenotypes. Tissue- and/or time-specific reversion of the mutation attained by breeding gene trap mutants to lines expressing CreER or CreERT2 in a tissue-specific manner would enable dissection of spatiotemporal requirement of the trapped gene. This functionality gives mutant alleles made with GBT-B1 an advantage over alleles made chemically, with various targeted nucleases, or with retrovirus. However, it would be even more beneficial to combine the high mutagenicity of GBT-B1 (perhaps improved to GBT-RP2 levels) with the full conditionality offered by two-recombinase inversion systems such as FleX, recently adapted for use in zebrafish [70,71]. Combination of additional functionality offered by Gal4-VP16 with high mutagenicity and full conditionality would provide a very powerful tool for dissection of gene function in the zebrafish.

## Methods

### Construction of vectors

To build our gene trap vector and UAS:mRFP reporter transgenes, we used GBT-R15 [27,28], components of Gal4-VP16 vectors generated by the Fraser laboratory [34], miniTol2 [37], lens-specific GFP expression cassette γCry:GFP [19] and monomeric Red Fluorescent Protein (mRFP) [76]. Details on vector construction are available upon request. The gene trap construct GBT-B1 (*G*ene *B*reaking *T*ransposon – *B*ipartite 1, pDB783) and Tol2/γCry:GFP,14XUAS:GFP (pDB790) are available from Addgene. To produce GBT-B2, the VP16 transcriptional activation domain was replaced by VP16-FF by PCR.

### Transgenesis procedures

Gene trap construct GBT-B1 (pDB783) was purified using Qiagen miniprep protocol including the optional PB wash step. Plasmid DNA (25 pg) was co-injected with 25 pg of Tol2 RNA into 1-cell zebrafish embryos in standard Tol2 transgenesis protocol [37,77]. Embryos injected with GBT-B1 were screened on Zeiss Axioscope or Zeiss Axioimager (both with 5X Fluar objective) for high levels of GFP fluorescence at 3 dpf. Approximately 20-30% of embryos surviving to day 3 were considered high expressors and raised to adulthood. The same methods were applied to generate the *Tg(Tol2/14XUAS:mRFP)* line, embryos were injected with pTol2/14XUAS:mRFP (pDB788) with Tol2 transposase mRNA and raised to adulthood. Adults were incrossed and embryos were screened for leaky expression of mRFP. A single transgenic line was established. To produce *Tg(Tol2/gCry:GFP,14XUAS:mRFP)tpl2* line, we injected embryos with Tol2 mRNA and Tol2/gCry:GFP, 14XUAS:mRFP (pDB790), screened for GFP expression in the lens and raised to adulthood. Adults were incrossed and embryos were screened for lens-specific GFP expression. A single transgenic line was established.

### Inverse PCR

Genomic DNA was prepared from batches of 20 GFP positive and GFP-negative embryos from an F1 or F2 outcross. In separate reactions, the genomic DNA was digested with NlaIII, TaqI, NheI/SpeI/XbaI/XmaJI or BamHI/BclI/BglII, then diluted and ligated overnight as described in [28,78]. Several different primer combinations were used in inverse PCR. For most lines, the first PCR reaction was carried out using primers Tol2-F13 and Tol2-R4 (primer sequences are listed in Additional file 9: Table S1). 0.1-1uL of the first PCR was used to carry out the second PCR reaction using primers Tol2-F11 and Tol2-R5. To identify genes mutated in *tpl17*, *tpl23* and *tpl25*, two sets of inverse PCR reactions were carried out. For 5’ end of the gene trap, first PCR was carried out with Tol2-F8 and B1/5’No3 and the second PCR was carried out with Tol2-F4 and B1/5’No2. For 3’ end of the trap, the first PCR was carried out with Tol2-R3 and B1/3’No1 and the second PCR was carried out with Tol2-R4 and B1/3’No2.

### Identification of Gal4 fusion transcripts by 5’RACE

Total RNA was prepared from a pool of 20 GFP positive and a pool of 20 GFP negative 5 dpf embryos using Absolutely RNA miniprep kit (Agilent Technologies, Cat# 400800). cDNA was made from 250-500 ng of total RNA using SuperScript™ II Reverse Transcriptase (Invitrogen, Cat # 18064–022) following previously described protocol [28,79]. Race-ready cDNA was amplified by PCR using primer mix containing KJC-002 and KJC-003 at a ratio of 1:5 and Gal4-R2. Second PCR reaction was performed using either 1 μL of undiluted or 1 μL of 1:10 dilution of the first PCR, using KJC-004 and Gal4-R3 primers. PCR products from the second PCR reaction were run on 1% agarose gel, and bands obtained on GFP-positive embryo DNA but absent from GFP-negative siblings were purified using either Qiagen or Thermofisher Fermentas Gel Extraction kits and sequenced.

### Confirmation of Gal4 mRNA fusion by RT-PCR

Once gene trap integration linked to GFP expression was identified by iPCR (or 5’RACE), the presence of Gal4 mRNA fusion was confirmed by RT-PCR. For this, cDNA was made from 250-500ng of total RNA prepared from a pool of GFP positive 5 dpf embryos using SuperScript™ III Reverse Transcriptase (Invitrogen, Cat # 18080–044) and random hexamers following protocol supplied by the manufacturer. 1 μl of the cDNA was used for PCR amplification using forward genomic primer for an exon upstream of the integration and a reverse primer for Gal4 (Gal4-R2 or Gal4-R3). PCR bands were sequenced with Gal4 reverse primer to confirm correct reading frame of the fusion mRNA.

### Southern Hybridization

Genomic DNA was prepared from a pool of 20 GFP positive and a pool of 20 GFP negative 5 dpf F2 generation embryos from 13 gene trap lines (*tpl3*, *tpl4*, *tpl5*, *tpl6*, *tpl7*, *tpl8*, *tpl9*, *tpl10*, *tpl11*, *tpl12*, *tpl13*, *tpl14*, *tpl15*). Genomic DNA (5 μg) was digested with HpaI and NdeI, subjected to 1% agarose gel electrophoresis and transferred onto Amersham Hybond™-XL (GE Healthcare, code RPN303S) membrane. Hybridization was carried out with ^32^P-labelled eGFP probe (ca 700 bp) that was obtained by PCR amplification using original gene trap vector as a template.

For *tpl6* and *tpl10* lines, additional Southern analyses were performed on genomic DNA digested with HindIII, NsiI and HindIII/NsiI.

### Genotyping

Genotyping strategy was designed for gene trap insertions with known genomic integration sites. Genotyping was typically performed using three-primer PCR: forward genomic primer upstream of the integration, reverse genomic primer downstream of the integration, and a gene trap (Tol2) primer. Wild type allele produces an amplicon between two genomic primers, while the gene trap allele gives rise to a product between the gene trap primer and one of the genomic primers. Primers were selected so that wild type band and gene trap band would differ in size. For an example, see Additional file 7: Figure S5, lanes 17 and 18. Primer sequences are listed in Additional file 9: Table S1, and exact genotyping PCR conditions are available upon request. In cases where three-primer PCR failed, standard two primer PCR was performed using one transposon primer and one genomic primer on gene trap positive and gene trap negative embryo DNA.

### RT-qPCR

RT-qPCR was used to determine the efficiency of mRNA knockdown of insertionally mutated gene by quantifying relative amount of intact mRNA present in homozygous mutants versus wild type siblings. DNA and RNA were isolated from 3 GFP-negative and 10–15 GFP-positive 5 dpf embryos using Trizol reagent (Ambion). DNA fraction was used for genotyping, and RNA was used to prepare cDNA from 3 wild type and 3–4 homozygous mutant siblings. qPCR was carried out using LightCycler® 480 SYBR Green I Master kit (Roche, Cat # 04707516001) with genomic primers for trapped gene’s exons flanking the transposon integration. Each sample was analyzed in triplicates. β-actin was used as a “reference” gene to normalize for differences in cDNA yields. PCR conditions were as follows: 5 min initial incubation at 95°C, followed by 42 cycles of 95°C for 10 sec, 57°C for 15 sec and 72°C for 1 min, and ending with a final step at 72°C for 7 min. qPCR data were analyzed using LightCycler® 480 1.5 software. Expression levels of intact mRNA of the mutated gene (”target” gene) for each sample were calculated as ratio of CT values for “target” and “reference” genes, averaged over triplicates assuming equal PCR amplification efficiencies (=normalized expression) [80]. Genotyping and qPCR primers are listed in Additional file 9: Table S1.

### *In situ* hybridization

Analysis of co-expression of endogenous *nsfa* transcript with *eGFP* was performed by whole-mount *in situ* hybridization on 1 dpf embryos as described previously [50]. *nsfa* full-length cDNA was obtained by PCR amplification using primers nsf/kpn-F1 and nsf/Cla-R1 and 5 dpf embryo cDNA as the template. The 2.2 kb PCR band was cloned into the pCR®II-TOPO® vector (Invitrogen K4600-01). Resulting plasmid was linearized with BamHI and riboprobe synthesis was performed using DIG RNA Labeling kit (Roche 11 175025910) with T7 RNA polymerase in 20 μL of reaction volume. The reaction was carried out at 37°C for 2 h, and incubated for additional 15 min with 2 μl of DNase I at the same temperature. RNA was precipitated by adding 2 μL of 7.5 M Lithium Chloride and 75 μl of 100% ethanol following incubation at −20°C overnight. Following centrifugation, RNA pellets were washed with 70% ice-cold ethanol and dissolved in 30 μl of RNase-free water. eGFP probe was obtained by PCR amplification with ^eGFP-F1 and Cla/BGYFP-R1 (primer sequences are in Additional file 9: Table S1) using GBT-B1/pDB783 as a template. Probes for *ebf3*, *cyp26c1* and *snap25b* were PCR amplified from 5 dpf embryo cDNA using corresponding primers listed in Additional file 9: Table S1. All subsequent steps were the same as described for *nsfa* probe.

### Site-specific recombinases

Cre mRNA was transcribed using pT3TS/Cre (pDB638) as the template as described in [27,28]. For reversion of gene trap mutations, 25–75 pg of *in vitro* transcribed Cre mRNA was injected into 1-cell zebrafish embryos.

To synthesize Flp RNA, we first amplified eFLP coding sequence using primers Bgl-Flp-F1 and XbaCla-Flp-R1, with pCMV-Flpx9.MiDB [81] as the template. PCR product was cloned into pJet1.2 (ThermoFisher Fermentas) and then subcloned into pT3TS [82] to generate pDC31. pDC31 was linearized with BamHI and transcribed using mMessage Machine T3 *in vitro* transcription kit (Ambion). 600 pg of the *in vitro* transcribed eFLP mRNA was injected into the yolks of 1-cell stage zebrafish embryos in 3 nL volume.

## Abbreviations

cDNA: Copy DNA; cIMG: Candidate insertionally mutated gene; dpf: Days post fertilization; eGFP: Enhanced green fluorescent protein; GBT: Gene breaking transposon; hpf: Hours post fertilization; IMG: Insertionally mutated gene; iPCR: Inverse polymerase chain reaction; KV: Kupffers vesicle; mRFP: Monomeric red fluorescent protein; p(A): Polyadenylation and transcriptional termination sequence; PCR: Polymerase chain reaction; RT-PCR: Reverse transcription – Polymerase chain reaction; SA: Splice acceptor; UAS: Upstream activating sequence; 5’ RACE: 5’ rapid amplification of cDNA ends.

## Competing interests

The authors declare that they have no competing interests.

## Authors’ contributions

DB designed and constructed GBT-B1 gene trap vector and UAS:mRFP reporter. JB, DN, FB, DG, FB and DB carried out the insertional mutagenesis screen and analyzed recovered gene trap lines. JB, DN and DB identified insertionally mutated genes by inverse PCR and 5’ RACE. JB carried out quantitative PCR and analyzed the data. JB and DN carried out Southern Hybridization. JB and GB carried out *in situ* hybridization experiments. KW propagated gene trap lines and documented fluorescent reporter expression patterns. DC cloned eFlp recombinase for removal of UAS:eGFP. JB and DB wrote the manuscript. All authors read and approved the contents of the manuscript.

## Supplementary Material

Additional file 1: Figure S1 Comparison between embryos injected with GBT-B1 and GBT-B2 gene traps containing ^Gal4-VP16 and ^Gal4-FF respectively. Embryos were injected with Tol2 transposase mRNA and GBT-B1 (A) or GBT-B2 (B) plasmid DNA. At 3 dpf, random GFP-positive embryos were photographed under identical settings and images were processed identically. Click here for file

Additional file 2: Figure S2 Enhancer trapping by GBT-B1. Solid black boxes and lines denote exons, pale pink boxes and lines denote untranslated regions, orange box denotes an enhancer and dashed orange arrows indicate transcriptional activation by the enhancer. Click here for file

Additional file 3: Figure S3 Expression of *nsfa* in the pancreas. Comparison between GFP expression of *nsfa*^*tpl6*^ gene trap **(A, C)** and *nsfa* expression by whole mount *in situ* hybridization **(B, D)** in 1 dpf zebrafish embryos. Red arrow points to pancreas. Click here for file

Additional file 4: Figure S4 Phenotypes of *nsf* ^*tpl6*^*and flr*^*tpl19*^ homozygotes. **A.** Larvae homozygous for *nsf*^ *tpl6*^ fail to inflate swim bladders by 6 dpf and display greatly reduced sensitivity to touch (Additional file 5: Movie 1). **B.** Comparison between *a flr*^*tpl19*^ homozygote and a wild type sibling at 3 dpf. Insert displays greater magnification of the kidney area with a cyst (white arrow). Click here for file

Additional file 5: Movie 1 Reduced touch response of *nsfa*^*tpl6*^ gene trap homozygotes. Embryos homozygous for *nsfa*^*tpl6*^ gene trap display very limited touch response. Click here for file

Additional file 6: Movie 2 Phenotype of *flr*^*tpl19*^ homozygotes. Embryos homozygous for *flr*^*tpl19*^ gene trap display abnormal body curvature and other abnormalities. Click here for file

Additional file 7: Figure S5 Late larval lethality of *atp1a3a*^*tpl10*^ homozygotes. A. 5 dpf larvae homozygous for *atp1a3a*^*tpl10*^ do not display overt embryonic phenotypes compared to heterozygous and wild type embryos. B. Genotyping of adult fish raised from *atp1a3a*^*tpl10*^ incross embryos selected for GFP fluorescence. Diagrams on the top represent the *tpl10* gene trap allele and wild type allele with expected sizes of PCR bands indicated. Genomic primers flanking transposon integration (Atp1a3a.9A1c.F and Atp1a3a.9A1A.R) are depicted as blue arrows, transposon-specific primer Tol2-R5 is depicted as a black arrow. PCR bands are shown as blue (wild type) and black (gene trap allele) dashed lines. Short PCR extension time does not allow amplification across GBT-B1. Below the diagram is a picture of a genotyping gel. Lanes 1 an 16, DNA ladder (Thermofisher Fermentas Cat #SM0331, sizes of relevant bands indicated to the left). Lanes 2–15 are PCR reactions on DNA from individual tailclips. Lanes 17 and 18 are PCR reactions performed on DNA from pools of GFP-positive (lane 17) and GFP-negative (lane 18) embryos. Click here for file

Additional file 8: Table S2 Germline excision of UAS:eGFP cassette from *nsf*^*tpl6*^ gene trap line. Gene trap heterozygotes were crossed to *Tg(UAS:mRFP)tpl2* and embryos were injected with 600 pg of *in vitro* transcribed Flp mRNA. Embryos with significant reduction of eGFP expression were raised to adulthood and crossed to *Tg(UAS:mRFP)tpl2* again. Column 1, fish identifier. Column 2, number of embryos obtained. Column 3, total number of RFP-positive embryos. Column 3, number of RFP-positive embryos not expressing eGFP. Column 3, calculated efficiency Flp-mediated eGFP excision in germline. Click here for file

Additional file 9: Table S1 Primer sequences. Click here for file
