# Supplementary figures and images for: Efficient disruption of Zebrafish genes using a Gal4-containing gene trap
Source: BMC Genomics. 2013 Sep 14;14:619. doi: 10.1186/1471-2164-14-619 (PMC3848861; doi:10.1186/1471-2164-14-619)

## Slide 1
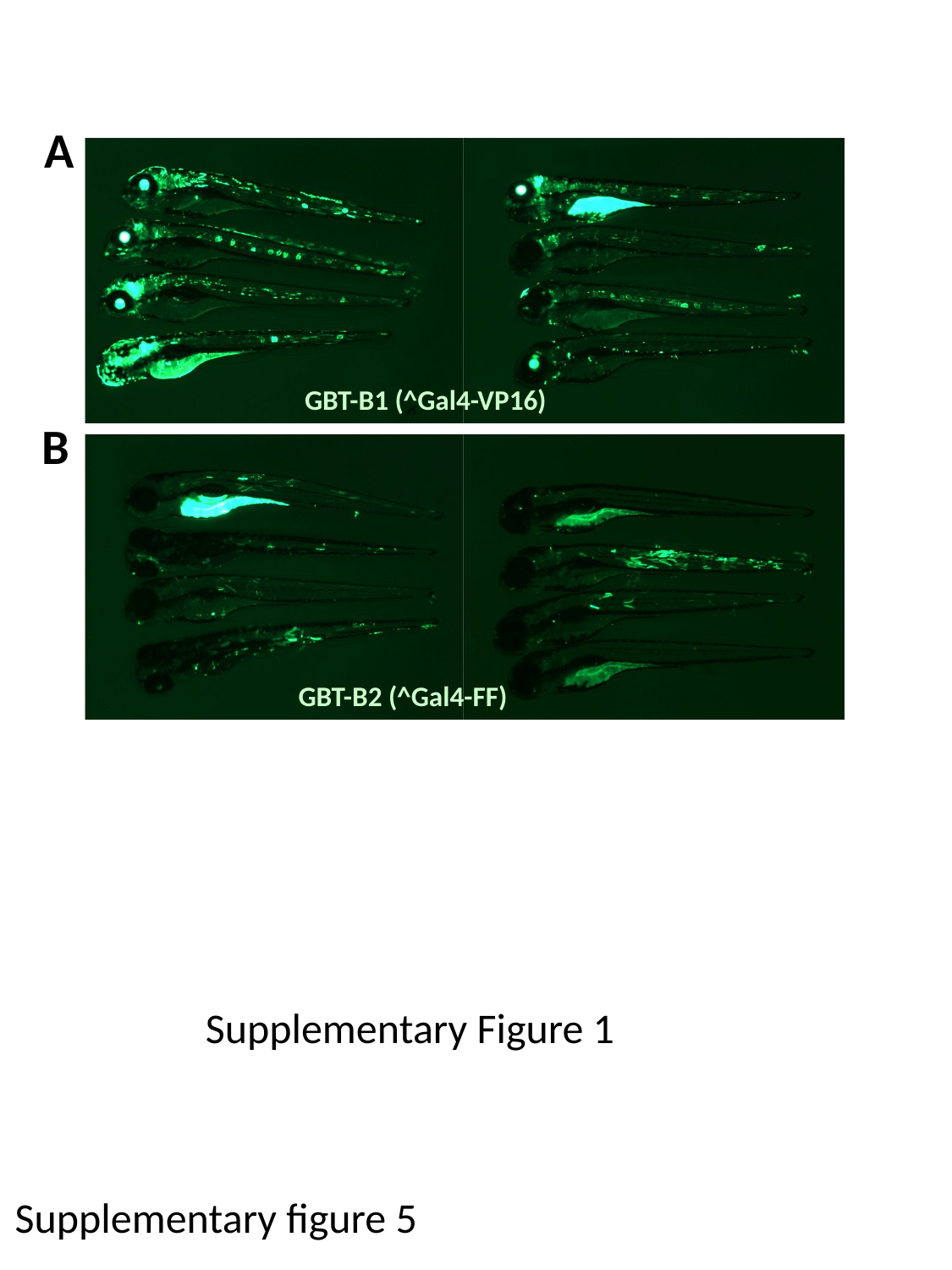

A
 GBT-B1 (^Gal4-VP16)
B
GBT-B2 (^Gal4-FF)
Supplementary Figure 1
D
Supplementary figure 5

Supplement: Additional file 1: Figure S1 — Comparison between embryos injected with GBT-B1 and GBT-B2 gene traps containing ^Gal4-VP16 and ^Gal4-FF respectively. Embryos were injected with Tol2 transposase mRNA and GBT-B1 (A) or GBT-B2 (B) plasmid DNA. At 3 dpf, random GFP-positive embryos were photographed under identical settings and images were processed identically. [file 1471-2164-14-619-S1.pptx]

## Slide 1
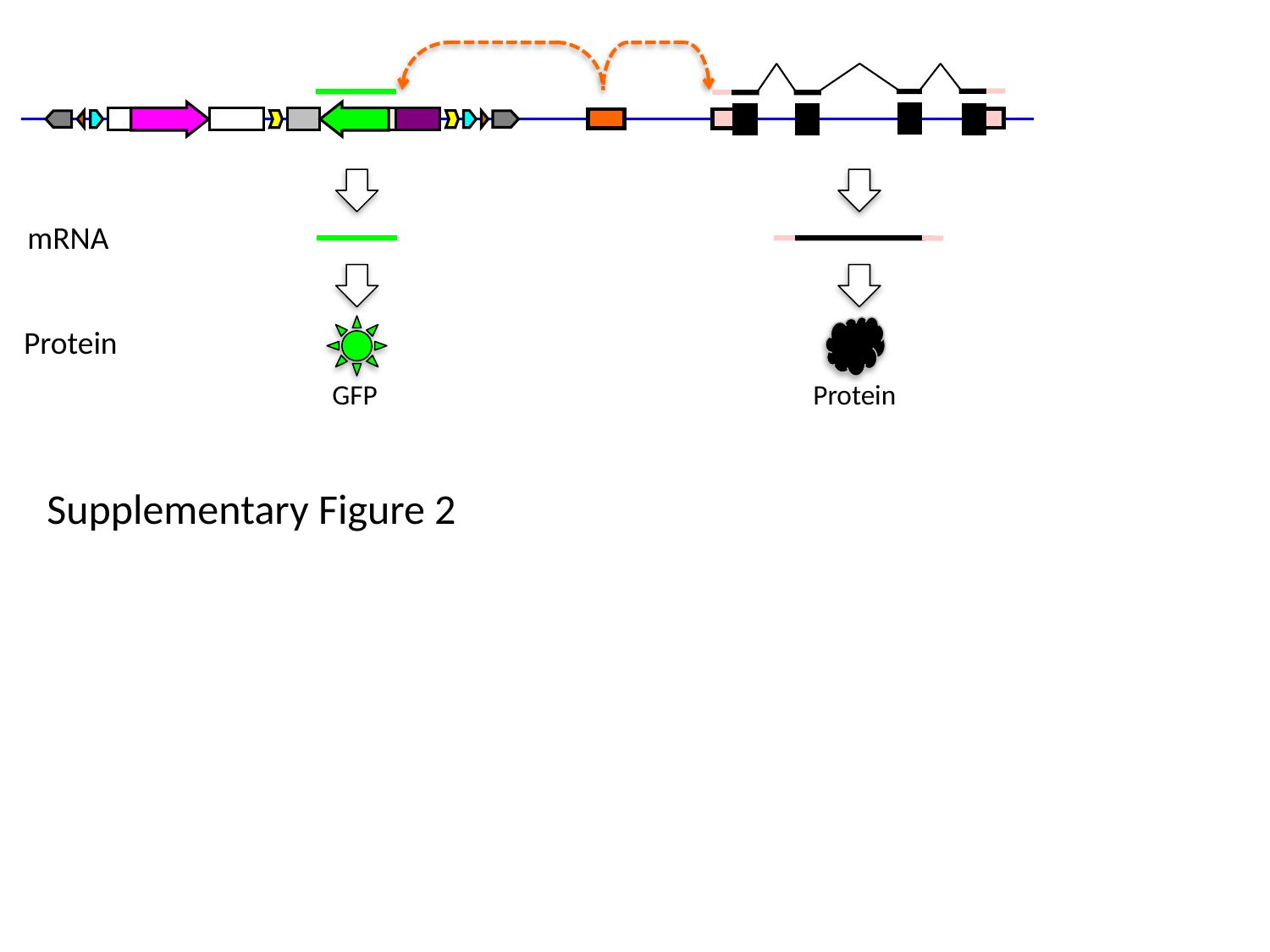

mRNA
Protein
GFP
Protein
Supplementary Figure 2

Supplement: Additional file 2: Figure S2 — Enhancer trapping by GBT-B1. Solid black boxes and lines denote exons, pale pink boxes and lines denote untranslated regions, orange box denotes an enhancer and dashed orange arrows indicate transcriptional activation by the enhancer. [file 1471-2164-14-619-S2.pptx]

## Slide 1
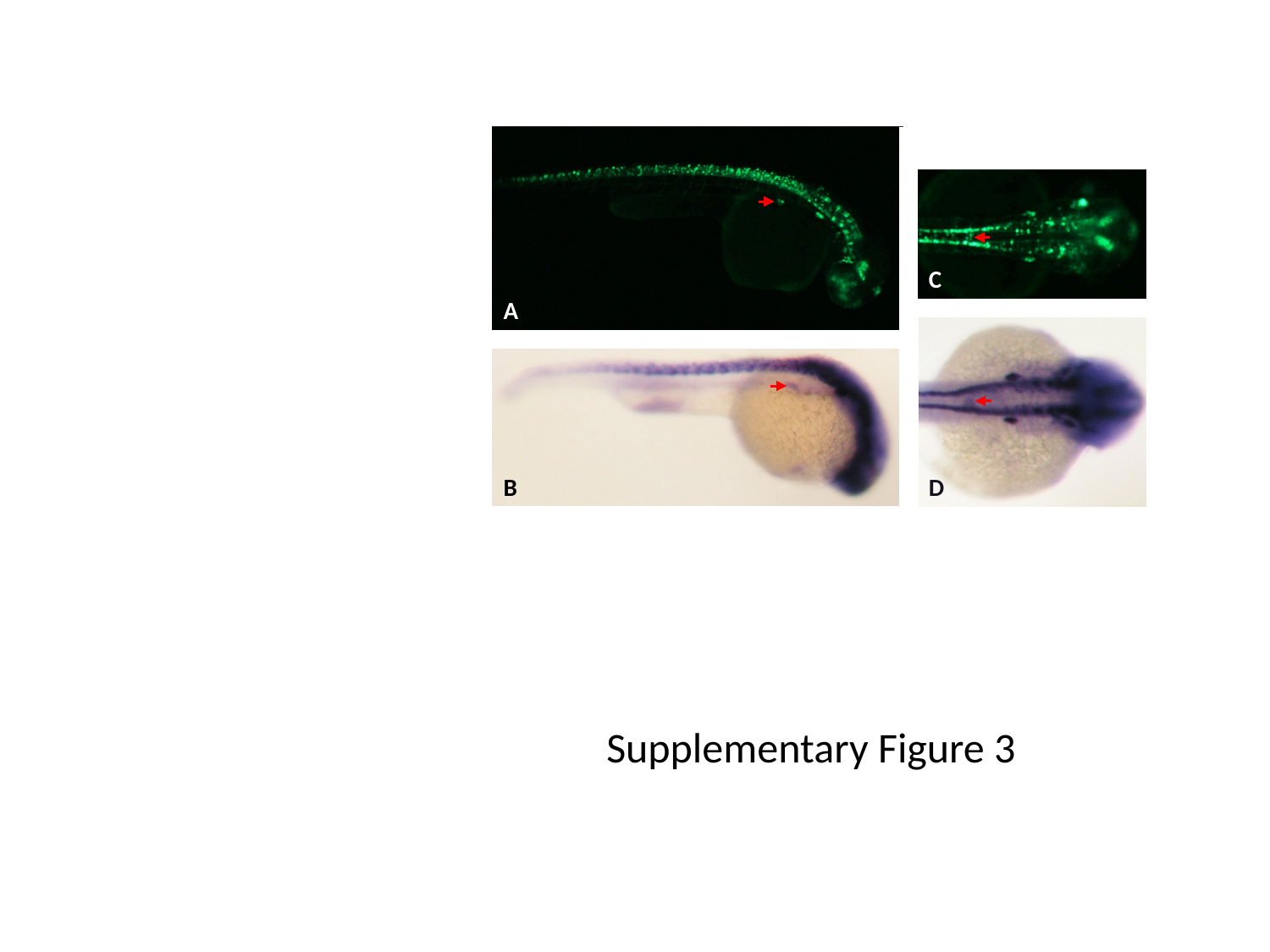

C
A
B
D
Supplementary Figure 3

Supplement: Additional file 3: Figure S3 — Expression of nsfa in the pancreas. Comparison between GFP expression of nsfatpl6 gene trap (A, C) and nsfa expression by whole mount in situ hybridization (B, D) in 1 dpf zebrafish embryos. Red arrow points to pancreas. [file 1471-2164-14-619-S3.pptx]

## Slide 1
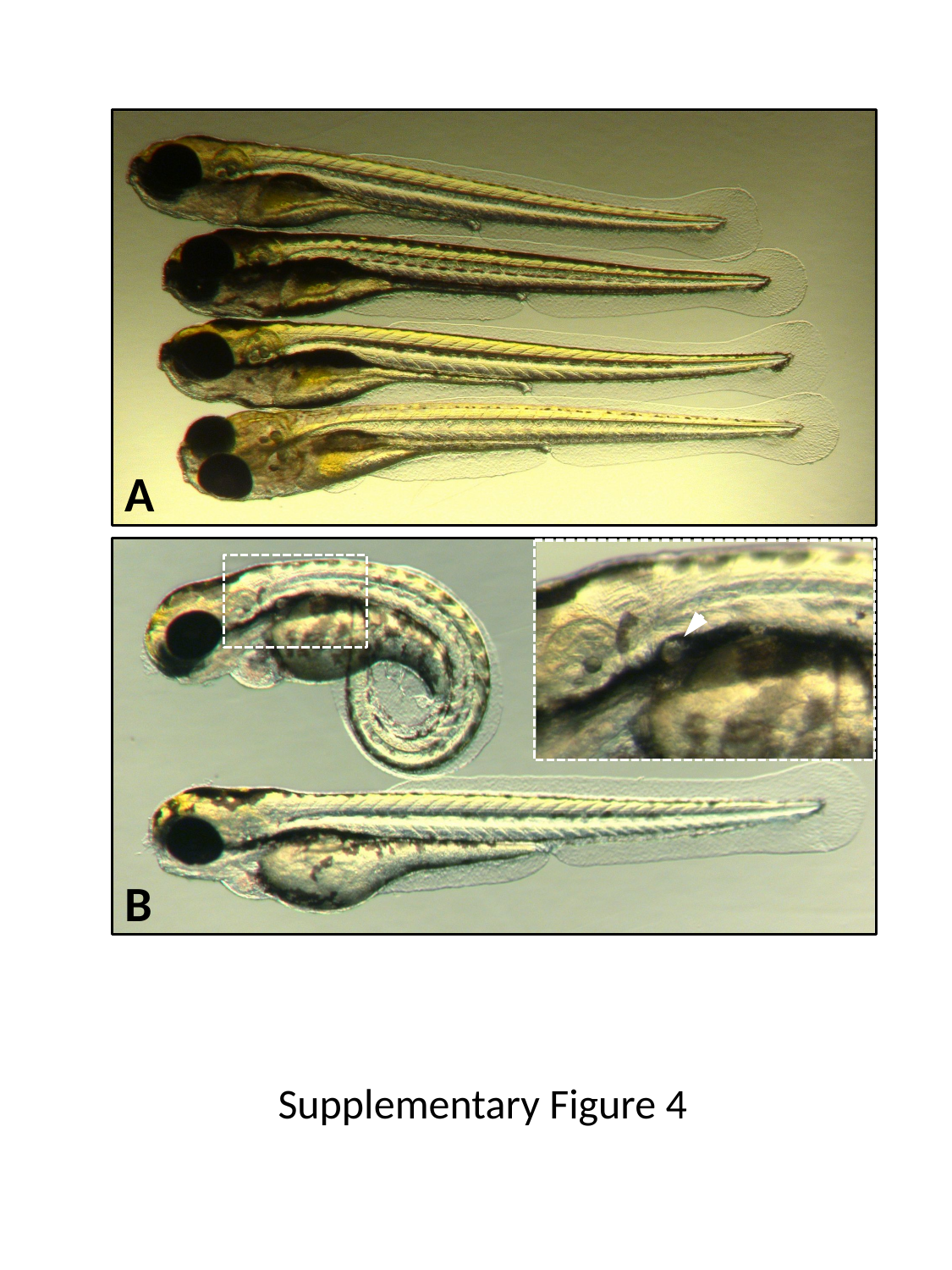

A
B
Supplementary Figure 4

Supplement: Additional file 4: Figure S4 — Phenotypes of nsf tpl6and flrtpl19 homozygotes. A. Larvae homozygous for nsf tpl6 fail to inflate swim bladders by 6 dpf and display greatly reduced sensitivity to touch (Additional file 5: Movie 1). B. Comparison between a flrtpl19 homozygote and a wild type sibling at 3 dpf. Insert displays greater magnification of the kidney area with a cyst (white arrow). [file 1471-2164-14-619-S4.pptx]

## Slide 1
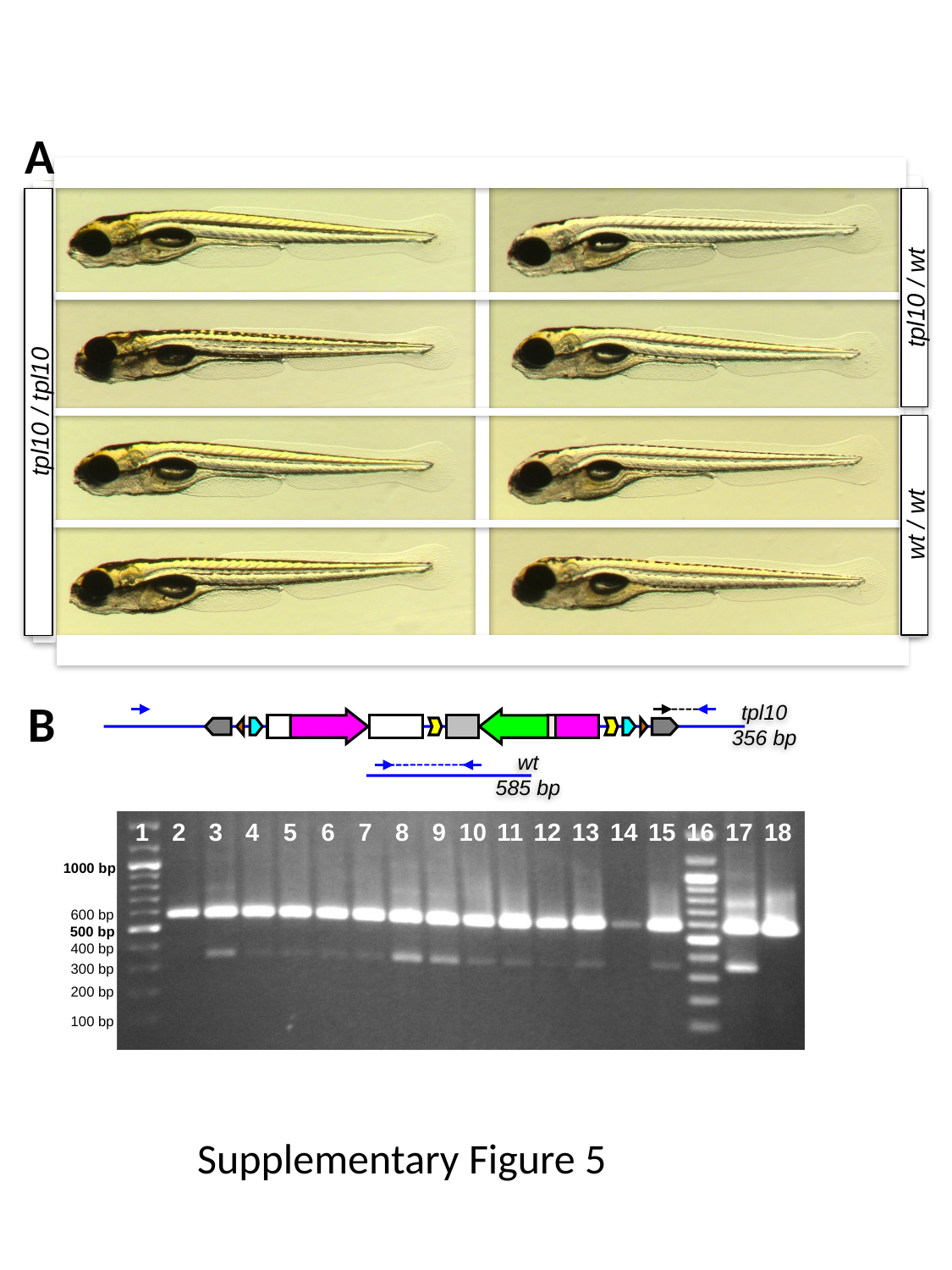

A
tpl10 / wt
tpl10 / tpl10
wt / wt
B
tpl10
356 bp
wt
585 bp
1
2
3
4
5
6
7
8
9
10
11
12
13
14
15
16
17
18
1000 bp
600 bp
500 bp
400 bp
300 bp
200 bp
100 bp
Supplementary Figure 5

Supplement: Additional file 7: Figure S5 — Late larval lethality of atp1a3atpl10 homozygotes. A. 5 dpf larvae homozygous for atp1a3atpl10 do not display overt embryonic phenotypes compared to heterozygous and wild type embryos. B. Genotyping of adult fish raised from atp1a3atpl10 incross embryos selected for GFP fluorescence. Diagrams on the top represent the tpl10 gene trap allele and wild type allele with expected sizes of PCR bands indicated. Genomic primers flanking transposon integration (Atp1a3a.9A1c.F and Atp1a3a.9A1A.R) are depicted as blue arrows, transposon-specific primer Tol2-R5 is depicted as a black arrow. PCR bands are shown as blue (wild type) and black (gene trap allele) dashed lines. Short PCR extension time does not allow amplification across GBT-B1. Below the diagram is a picture of a genotyping gel. Lanes 1 an 16, DNA ladder (Thermofisher Fermentas Cat #SM0331, sizes of relevant bands indicated to the left). Lanes 2–15 are PCR reactions on DNA from individual tailclips. Lanes 17 and 18 are PCR reactions performed on DNA from pools of GFP-positive (lane 17) and GFP-negative (lane 18) embryos. [file 1471-2164-14-619-S7.pptx]
