# Supplementary material for: Efficient disruption of Zebrafish genes using a Gal4-containing gene trap
Source: BMC Genomics. 2013 Sep 14;14:619. doi: 10.1186/1471-2164-14-619 (PMC3848861; doi:10.1186/1471-2164-14-619)
Supplement: Additional file 8: Table S2 — Germline excision of UAS:eGFP cassette from nsftpl6 gene trap line. Gene trap heterozygotes were crossed to Tg(UAS:mRFP)tpl2 and embryos were injected with 600 pg of in vitro transcribed Flp mRNA. Embryos with significant reduction of eGFP expression were raised to adulthood and crossed to Tg(UAS:mRFP)tpl2 again. Column 1, fish identifier. Column 2, number of embryos obtained. Column 3, total number of RFP-positive embryos. Column 3, number of RFP-positive embryos not expressing eGFP. Column 3, calculated efficiency Flp-mediated eGFP excision in germline. [file 1471-2164-14-619-S8.docx]

|  | N= | RFP+ | RFP+/GFP- | % excised |
| --- | --- | --- | --- | --- |
| Fish 1 | 91 | 49 | 49 | 100% |
| Fish 2 | 15 | 7 | 7 | 100% |
| Fish 3 | 56 | 27 | 27 | 100% |
| Fish 4 | 7 | 4 | 4 | 100% |
| Fish 5 | 15 | 8 | 3 | 37.5% |
| Fish 6 | 55 | 32 | 32 | 100% |

**Supplementary Table 2**. Germline excision of UAS:eGFP cassette from *nsf^tpl6^* gene trap line. Gene trap heterozygotes were crossed to *Tg(UAS:mRFP)tpl2* and embryos were injected with 600 pg of *in vitro* transcribed Flp mRNA. Embryos with significant reduction of eGFP expression were raised to adulthood and crossed to *Tg(UAS:mRFP)tpl2* again. Column 1, fish identifier. Column 2, number of embryos obtained. Column 3, total number of RFP-positive embryos. Column 3, number of RFP-positive embryos not expressing eGFP. Column 3, calculated efficiency Flp-mediated eGFP excision in germline.
