# Supplementary material for: Efficient disruption of Zebrafish genes using a Gal4-containing gene trap
Source: BMC Genomics. 2013 Sep 14;14:619. doi: 10.1186/1471-2164-14-619 (PMC3848861; doi:10.1186/1471-2164-14-619)
Supplement: Additional file 9: Table S1 — Primer sequences. [file 1471-2164-14-619-S9.docx]

|  |  | **General Primers** |
| --- | --- | --- |
|  |  |  |
|  | Primer name | Sequence 5’ to 3’ |
|  |  |  |
|  | Tol2-F13 | GTACTTATTTTTTGGAGATCACTTC |
|  | Tol2-R4 | ATAATACTTAAGTACAGTAATCAAG |
|  | Tol2-F11 | CCCTTGCTATTACCAAACCAATTGA |
|  | Tol2-R5 | TAATCAAGTAAAATTACTCAAGTAC |
|  | KJC-002 | AATGGCTTCCGAGACCTGCTGTCCAAGTTCGTACAAAAAAGCAGGC |
|  | KJC-003 | AATGGCTTCCGAGACCTGCTGT |
|  | Gal4-R2 | TTCTTCAGACACTTGGCGCACTTCG |
|  | KJC-004 | GAGACCTGCTGTCCAAGTTCGT |
|  | Gal4-R3 | TAAGTCGGCAAATATCGCATGCTTG |
|  |  |  |
|  |  |  |
|  | Bgl-Flp-F1 | AGATCTACCATGCCACAATTTGGTATATTATGTA |
|  | XbaCla-Flp-R1 | TCTAGAATCGATTATATGCGTCTATTTATGTAGGA |
|  | B1/5’No3 | CTAGAGATTCTTGTTTAAGCTGTAG |
|  | B1/5’No2 | TGTATGCTATACGAAGTTATCAGCA |
|  | B1/3’No1 | CAGGGTAATATAACTTCGTATAGCA |
|  | B1/3’No2 | GCATACATTATACGAAGTTATCGTT |
|  | Bgl-Flp-F1 | AGATCTACCATGCCACAATTTGGTATATTATGTA |
|  | XbaCla-Flp-R1 | TCTAGAATCGATTATATGCGTCTATTTATGTAGGA |
|  |  |  |
|  |  | **Genotyping Primers** |
|  |  |  |
| Line | Primer name | Sequence 5’ to 3’ |
|  |  |  |
| tpl3 |  | Integration site not determined |
| tpl3 |  | Integration site not determined |
| tpl4 | zStat5.1_ex10.1F |  |
| tpl4 | zStat5.1_ex11.1R |  |
| tpl5 | 7A1c.F | TGATTTGAGCATGGAAGTGTG |
| tpl5 | 7A1B.R | TGTCCACCAAGCCATCAGTA |
| tpl6 | Nsf.1f | CGTGATGTATAACCTTGACG |
| tpl6 | Nsf.2f | GCGGTTGATTGCTCACTTAT |
| tpl6 | Nsfint1.2R | AACATTCAGAAGATACAAGC |
| tpl7 | Jam3b | Forward primer was not designed |
| tpl7 | Jam3.8A2B.R | CATGAGACTTTAGGAGGATTTTCA |
| tpl8 | zfp36I2_ex1.1F | CAACGATTGGAAGATGACCA |
| tpl8 | zfp36I2_ex2.1R | AACTGAGGATGTTATTATTG |
| tpl9 |  | Integration site not determined |
| tpl9 |  | Integration site not determined |
| tpl10 | Atp1a3a.9A1c.F | GCTAGTTGGAAAGGCGACAT |
| tpl10 | Atp1a3a.9A1A.R | TGATATGGCCTTGCGTTACA |
| tpl10 | Atp1a3a.9A1exr.1 | TGTCCTCGACTGTCTCGTTG |
| tpl11 | Bbs7_int3.1F | tagcatcacgcagttgcaga |
| tpl11 | Bbs7.9A2a.R | AGCTCCTCCAAGCTCAAGTCT |
| tpl12 | 9A3_SGIP_int6.2F | actcttctgtcagtgtgctctca |
| tpl12 | 9A3b_SGIP.R | GCATATGAAGTGCGAATCCTG |
| tpl13 | 9A4c.F | CTGTGCTGAAGAAACGTCCA |
| tpl13 | 9A4b.R | gccatcagttctgactttgga |
| tpl14 | 9B1exf.1 | tctggcttccaacacagtga |
| tpl14 | 9B1.R | cagcagtttgtctgcatgct |
| tpl15 |  | Integration site not determined |
| tpl15 |  | Integration site not determined |
| tpl16 | EBFint7.1F | AATAGGGAGGAGGAGGAGTCTG |
| tpl16 | EBFint7.1R | TGGAGGTGGCATTTAGCTTAG |
| tpl17 | PlecbInt-F1 | TCTATAGGAAGCAGCTTTACAATAT |
| tpl17 | PlecbInt-R1 | GCTCATTCTGACCTCTGCCTGTCTT |
| tpl18 | Fam46utr-F1 | TGGAGACGGAGACGCCTGAGTTCAT |
| tpl18 | Fam46-R2 | GTGCCAAATGGGTTAAATAAGCATG |
| tpl18 | Fam46atg-F1 | ATGTCCACCGCGGATGTGTCGGAGC |
| tpl19 | FlrUTR-F1 | GCGTCATGTATTGATTACGTCACT |
| tpl19 | FlrAtg-F1 | ATGATGTCTGCGTCGTTCCACTGA |
| tpl19 | Flrex3-R2 | GTCTTGCATGTGATAGTAACAGTAG |
| tpl20 | Lasp1int1-F1 | AGCTTTAGCTACTAATGTGTACC |
| tpl20 | Lasp1int1-R1 | TAGACCTGTCACGATAAGCGATT |
| tpl20 | Lasp1atg-F1 | ATGAACCCGCTGTGTAGCAGA |
| tpl21 | Triqk-F1 | TGCTGCGAGCCACTCGGCTGAA |
| tpl21 | Triqk-R1 | GAGCTTCTCCTCTGAGAGTCAAAC |
| tpl22 | Baiapint-F1 | CTGATCTGTCTGCGAGGTTGCAA |
| tpl22 | Baiapint-R1 | CCTTGCATGATCGATTACAGGAT |
| tpl22 | Baiapatg-F1 | AATGTCTCGAGCCTCGGAGGAC |
| tpl23 | FNBP1int-F1 | GCTTACGTAATATGCAGTAGTTCA |
| tpl23 | FNBP1ex-R1 | GGGACGTTAACAGGGACATAAG |
| tpl24 | Cyp26c1_ex3.1F | AGACGAGAGAAACATGGAAAGG |
| tpl24 | Cyp26c1_ex4.2R | TAAGTGATTTGCTGCTCCTCC |
| tpl25 | Srp68ex4-F1 | CAAACATGGAGACCAGCTGGAGA |
| tpl25 | Srp68ex5-R1 | CCAGTCAGATAAGCAGTGTAAGC |
| tpl26 | Fam89ex-F1 | GGACATGGAGAGGATGTACGTGA |
| tpl26 | Fam89in-R1 | CGGAACTAATCTGTGATGCTCAGA |
| tpl27 | snap25b_ex4.1F | AGAACTTTGGTTATGCTGGACG |
| tpl27 | snap25b_int4.1R | CCTTAAATGATCCCGCCACC |
|  |  |  |
|  |  | **qPCR primers** |
|  |  |  |
|  | Primer name | Sequence 5’ to 3’ |
|  |  |  |
|  | *zfp36l2-F* | caacgattggaagatgacca |
|  | *zfp36l2-R* | GATGGATCTGCCTTAGCAATGTTC |
|  | nsfa-F | TGAGGAGCTCAACCGAGCAG |
|  | nsfa-R | CAGGGAGCACAGAGTGGTGA |
|  | *atp1a3a-F* | cagcttggcccattctttacg |
|  | *atp1a3a-R* | GCAGCCCTGGCATTAGTCAGA |
|  | stat5.1-F | cgacagcagtggcgagatct |
|  | stat5.1-R | tggattcacactggagctgc |
|  | bbs7-F | ATGATGGGGTTGTGAGCTGT |
|  | bbs7-R | GTCTCCACGGGTAAACACAGA |
|  | *nov*Oxy-F | TCTGGCTTCCAACACAGTGA |
|  | *nov*Oxy-R | ATGACGCTCCTCTGGTCATC |
|  | Sgip1-F | GGAGGATCACCGCAAAAAGTTTA |
|  | Sgip1-R | cttcactggtcttctgctcatc |
|  | lasp1-F | ATGAACCCGCTGTGTAGCAGA |
|  | lasp1-R | TGTTGTTTCAGACGCAGGTTC |
|  |  |  |
|  |  | **PCR rimers for *in situ* probes** |
|  |  |  |
| eGFP | Cla/BGYFP-R1 | ATCGATTACTTGTACAGCTCGTCC |
| eGFP | ^eGFP-F1 | AGGTACCGATATCTGGTGAGCAAGGGCGAGG |
| nsfa | Nsf/Kpn-F1 | AGGTACCATGGCGACCCGGACGATGC |
| nsfa | Nsf/Cla-R1 | TATCGATTACTCGAAGAAGCCGCGGT |
| snap25b | Sanp25b_Utr.F2 | GTTCATCAAGTGCAAGCTTTCAAGT |
| snap25b | Snap25b UTR.R2 | GCGCTACGTCTCAATATGAAGCATG |
| cyp26c1 | Cyp26c1_ATg.F1 | ATGTTCGGGCACGATTTCTGCCTCG |
| cyp26c1 | Cyp26c1-Taa.R1 | GCTTATATGTGCGTGGACTCCCTC |
| ebf3 | Ebf3_ORf. F1 | CAGCCAGTGGAGATCGAAAGGACAG |
| ebf3 | Ebf3_ORf. R1 | TGCCGTAGGGAGAGTTCGCAGAGGA |
|  |  |  |
|  |  |  |
|  |  |  |

**Supplementary Table 1. Primer sequences.**
